# Analysis of Poly(ethylene terephthalate) degradation kinetics of evolved *Is*PETase variants using a surface crowding model

**DOI:** 10.1016/j.jbc.2024.105783

**Published:** 2024-02-22

**Authors:** En Ze Linda Zhong-Johnson, Ziyue Dong, Christopher T. Canova, Francesco Destro, Marina Cañellas, Mikaila C. Hoffman, Jeanne Maréchal, Timothy M. Johnson, Maya Zheng, Gabriela S. Schlau-Cohen, Maria Fátima Lucas, Richard D. Braatz, Kayla G. Sprenger, Christopher A. Voigt, Anthony J. Sinskey

**Affiliations:** 1 Department of Biology, Massachusetts Institute of Technology, Cambridge, Massachusetts, USA; 2 Department of Chemical and Biological Engineering, University of Colorado, Boulder, Colorado, USA; 3 Department of Chemical Engineering, Massachusetts Institute of Technology, Cambridge, Massachusetts, USA; 4 Zymvol Biomodeling SL, Barcelona, Spain; 5 Department of Chemistry, Massachusetts Institute of Technology, Cambridge, Massachusetts, USA; 6 AgroParisTech, Palaiseau, France; 7 Plasma Science and Fusion Center, Massachusetts Institute of Technology, Cambridge, Massachusetts, USA; 8 Department of Biological Engineering, Massachusetts Institute of Technology, Cambridge, Massachusetts, USA

**Keywords:** *Is*PETase, PETase, biochemical model, surface crowding, kinetics, PET biodegradation

## Abstract

Poly(ethylene terephthalate) (PET) is a major plastic polymer utilized in the single-use and textile industries. The discovery of PET-degrading enzymes (PETases) has led to an increased interest in the biological recycling of PET in addition to mechanical recycling. *Is*PETase from *Ideonella sakaiensis* is a candidate catalyst, but little is understood about its structure-function relationships with regards to PET degradation. To understand the effects of mutations on *Is*PETase productivity, we develop a directed evolution assay to identify mutations beneficial to PET film degradation at 30 °C. *Is*PETase also displays enzyme concentration-dependent inhibition effects, and surface crowding has been proposed as a causal phenomenon. Based on total internal reflectance fluorescence microscopy and adsorption experiments, *Is*PETase is likely experiencing crowded conditions on PET films. Molecular dynamics simulations of *Is*PETase variants reveal a decrease in active site flexibility in free enzymes and reduced probability of productive active site formation in substrate-bound enzymes under crowding. Hence, we develop a surface crowding model to analyze the biochemical effects of three hit mutations (T116P, S238N, S290P) that enhanced ambient temperature activity and/or thermostability. We find that T116P decreases susceptibility to crowding, resulting in higher PET degradation product accumulation despite no change in intrinsic catalytic rate. In conclusion, we show that a macromolecular crowding-based biochemical model can be used to analyze the effects of mutations on properties of PETases and that crowding behavior is a major property to be targeted for enzyme engineering for improved PET degradation.

Poly(ethylene terephthalate) (PET) is a major plastic polymer composed of terephthalic acid (TPA) and ethylene glycol, which are joined by ester linkages. Due to the presence of the ester bonds in PET, it is more amenable to biodegradation and has become the most studied synthetic polymer for enzymatic depolymerization. *Ideonella sakaiensis* is a bacterium discovered in 2016 that consumes PET as a carbon source (1). It harbors the PET-degrading enzyme *Is*PETase, which is a member of the cutinase family and one of the fastest known enzymes for depolymerization of PET at room temperature (1). The enzyme releases soluble PET degradation products such as ethylene glycol, TPA, mono-2-hydroxyethyl terephthalate (MHET), bis(2-hydroxyethyl) terephthalate (BHET), and potentially oligomers (2). Many rational engineering efforts have been aimed at enhancing *Is*PETase thermostability and high temperature activity, and many directed evolution screening methods have been developed to identify variants with prolonged activity and/or better activity at elevated temperatures (3, 4, 5, 6, 7). The thermostability of *Is*PETase has thus been improved drastically to be on par with known thermostable PETases (also belonging mostly to the cutinase family) (3, 4, 5). However, little is known about the biochemical impact of these mutations, other than improvements in stability and increased product yield over a given period of time at higher temperatures. Biochemical analyses of *Is*PETase kinetics are particularly difficult due to a lack of kinetic models that can interpret the nonideal kinetic behaviors of *Is*PETase and also account for variables that stem from the solid nature of the substrate. Thus, the effects of most of the activity mutations that have been identified remain largely unknown.

Kinetic analysis is the standard method to study effects of mutations on catalytic activity. The kinetic models traditionally used for this purpose are the conventional Michaelis–Menten (^*conv*^MM) model, in conditions of substrate-site excess, and the inverse Michaelis–Menten (^*inv*^MM) model, in conditions of enzyme excess (8). However, *Is*PETase displays a concentration-dependent inhibition behavior where increasing enzyme concentrations eventually lead to decreased depolymerization rates of solid PET, particularly at its optimal reaction pH of 9 (1, 2, 9, 10). This phenomenon, which herein will be referred to as the inhibition effect, has also been observed in cellulases and poly(hydroxyalkanoate) (PHA) depolymerases, suggesting that it is a general behavior observed in enzymatic degradation of solid substrates. The inhibition effect makes it complicated to interpret biochemical properties using the MM kinetic models, which assume ideal saturation behaviors. To adapt the ^*inv*^MM model to *Is*PETase, studies have fit exclusively the increasing portion of the kinetic data with the ^*inv*^MM model (10). This approach cannot be used to explain the inhibition behavior exhibited by *Is*PETase nor predict the optimal enzyme concentration given a substrate concentration. Furthermore, it is unknown whether the fitted parameters represent true biochemical properties of the considered enzymes (11, 12).

The only known macroscopic kinetic model that captures the inhibition effect was proposed by Mukai *et al.* (1993) to describe the kinetic profiles of poly(hydroxybutyrate) film degradation by PHA depolymerases (13). According to the Mukai model, the degradation reaction requires the interaction between the catalytic domain of a surface-bound enzyme and an adjacent free substrate site, due to the multidomain nature of PHA depolymerases. As a result, in systems described by the Mukai model, the degradation rate eventually decreases as the site coverage increases, and the degradation reaction is completely halted at full site coverage. The model proposed by Mukai *et al.* for multidomain PHA depolymerases cannot be directly applied to explain the inhibition effect of single-domain depolymerases, such as *Is*PETase, which do not require two adjacent binding sites to form an active complex (9, 13). Furthermore, the model was incomplete in that it did not take into account the effect of substrate concentration on reaction rate (13). Finally, the model makes the assumption that enzyme concentrations are in excess of substrate sites, which is not always true for a given system.

The inhibition dynamics of the Mukai model can be loosely interpreted as a form of macromolecular crowding, where an enzyme requires enough physical space, in this case an adjacent binding site, to perform catalysis. Similarly, the coarse-grained simulation model developed by Ezaki *et al.* (2019) successfully described the inhibition effect for cellulase by accounting for the competition between the binding and catalytic domains for space on the substrate surface (11). Though the Ezaki model captures the inhibition effect, it requires as input single-molecule experimental rate constants, and it is not intended for fitting macroscopic kinetic data. Crowding phenomena have also been observed in protein adsorption experiments, where increasing protein adsorption on a surface results in transition (such as reorientation) to more compact states due to crowding effects (14, 15). Interestingly, Avilan *et al.* (2023) reported that the inhibition effect of *Is*PETase can be modulated by increasing the substrate surface area, which increases the enzyme concentration at which the inhibition effect is observed; the authors proposed that these observations are likely due to a surface crowding (SC) phenomenon (9).

More broadly, macromolecular crowding is a phenomenon where increasing concentrations of macromolecules decrease accessible volume and thus favor protein conformations that best compact into the available space (16, 17, 18). The phenomenon has been extensively investigated using molecular dynamics and coarse-grained simulations, which have shown that crowding causes larger reductions in enzyme activity for enzymes with larger conformational changes during catalysis (16, 18). Similarly, the intramolecular distances between residues of a mesophilic protein was reduced upon addition of crowding agents, suggesting a more compact conformation, whereas the thermophilic counterpart was invariant in the presence of crowding agents (19). Macromolecular crowding effects have been observed experimentally using inert crowding agents such as 100 g/L Ficoll 70 and 50 g/L bovine serum albumin (BSA), which suggests that a separation distance of *∼*10 nm between molecule centers results in crowding (20).

In this study, WT *Is*PETase and the previously developed thermostable TS-PETase variant (S121E, D186H, N233C, R280A, S282C) were evolved using random- and semi-rational–directed evolution for improved activity at 30 °C to obtain a set of mutations for biochemical analysis (2, 7). To investigate how and if the isolated mutations enhanced *Is*PETase catalytic activity, we develop a novel biochemical model based on macromolecular crowding for depolymerases that display inhibition effects. To support the SC hypothesis, *Is*PETase density on the PET surface is probed with total internal reflectance fluorescence microscopy and adsorption experiments. Furthermore, molecular dynamics simulations are performed to investigate *Is*PETase behavior under crowded and uncrowded conditions. Finally, using the SC model, we determine the catalytic rate and binding parameters of the evolved variants to gain mechanistic insights into the effects of the mutations on PET degradation and enzyme properties to be targeted for engineering.

## Results

### Directed evolution of *Is*PETase identifies mutations that enhance ambient temperature productivity and thermostability

**Figure 1:**
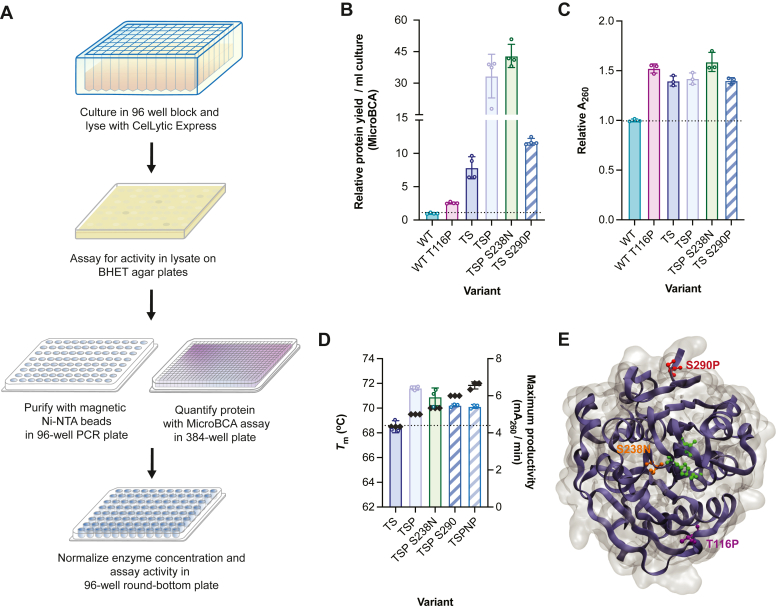
**Random- and semi-rational–directed evolution of *Is*PETase identified three mutations that enhancedpurification yields with potential benefits to activity.** Thatched *bars* represent mutations from *in silico* library. TSP is TS-T116P-PETase. *A*, schematic of high-throughput–directed evolution screening procedure. *B*, relative protein elution yields (based on MicroBCA quantification) of *Is*PETase variants in high-throughput validation screens. All yields were normalized to culture volume (per ml) and then to WT. Mean and SD are shown (*n* = 4 biological replicates shown in *circles*). *C*, relative product formation (A_260_) of hit mutants compared to WT based on high-throughput validation screens (2 h of incubation of semi-purified *Is*PETase variants with 0.25”-diameter amorphous Goodfellow PET films at 30 °C). Dashed lines indicate a fold-change of 1 for (*B*) and (*C*). *D*, melting temperature (*T*_m_, *black diamonds*) and maximum product formation rate (*bars*) of fully purified TS-PETase variants against 0.25”-diameter amorphous Goodfellow PET films at 30 °C. Initial rates of reaction were determined using the bulk absorbance assay and enzyme concentrations between 10 to 400 nM were assayed (Fig. S2*C*). Enzyme concentration at maximum product formation rate: TS-PETase = 100 nM, 80 nM for all other variants. Mean and SD of three biological replicates (*circles*) are shown for (*C*) and (*D*). *E*, positions of mutations identified from directed evolution are shown on the structure of WT (PDB: 6EQE) (24). The catalytic triad is shown in *green* as ball-and-stick model. *Purple*: T116P; *orange*: S238N; *red*: S290P. PET, poly(ethylene terephthalate).

An *in vitro*–directed evolution screen was developed for the evolution of PET-degrading enzymes on PET films. Variants of *Is*PETase with a C-terminal His_8_-tagged were expressed in *Escherichia coli*, lysed, and screened for activity on BHET-agar plates. All wells displaying activity were semi-purified using Ni-NTA resin, yielding sufficiently pure protein that can be quantified using MicroBCA and normalized to 100 nM for the final PET film activity assay (A_260_/A_280_ ratios of elutions suggested nucleic acid contamination but had minimal impact on A_260_ readings after normalization) (Fig. S1*B*). The reaction supernatants are then transferred to a UV-transparent 384-well plate and product levels are measured in a plate reader using A_260_ (Fig. 1*A*). The activity assay allows all abiotic conditions such as buffer composition, pH, temperature, and substrate type to be varied (see detailed methods in SI).

Using the described directed evolution assay, two random libraries and one *in silico* library were screened (total of 5400 variants). The selection conditions were 30 °C and 50 mM glycine-NaOH pH 9, 50 mM NaCl, and 10% v/v DMSO (herein referred to as reaction buffer) with 0.25"-diameter amorphous PET films as substrate (Goodfellow). WT *Is*PETase (WT) served as the template for the first random mutagenesis library, and several hit mutations were identified for known residues of interest such as R280S/H, Q119H, and N246D; these ultimately did not pass validation screens (Fig. S2, *A* and *B*) (7, 21, 22). A novel mutation, T116P, was found to increase PET degradation product accumulation and the amount of eluted protein in the validation screens (Fig. 1, *B* and *C*). Interestingly, this mutation had been previously considered in computational design and was not selected (5). T116P also increased the amount of eluted protein in validation screens when introduced into the thermostable variant TS-PETase (Fig. 1, *B* and *C*). The T116P mutation did not impact PET degradation in the TS-PETase background based on the screens, but kinetic assays of >99% purified TS-T116P-PETase (TSP-PETase) revealed a 30% improvement in maximum product accumulation rate compared to purified TS-PETase on 0.25”-diameter PET films at 30 °C (Figs. 1, *C* and *D*; S2*C* and S3). TSP-PETase was used as the template for the second round of random directed evolution, and S238N was shown to further increase eluted protein in the screens and potentially enhanced activity (Fig. 1, *B* and *C*). Many mutations have been identified at the S238 position with varying effects on product accumulation, substrate specificity, and thermostability (23, 24, 25, 26). Purified TSP-S238N-PETase had a cumulative 1.5 °C increase in thermostability compared to TS-PETase, though inclusion of S238N resulted in a small reduction in maximum productivity compared to TSP-PETase (Figs. 1*D* and S2*C*).

An *in silico* library consisting of 95 TS-PETase variants was also selected with the overarching objective of enhancing enzyme activity and thermostability (Fig. S2*D*). Mutation S290P was found to increase elution yield in screens with no effects on product accumulation (Fig. 1, *B* and *C*). This mutation has also been recently identified by Shi *et al.* (2023), further validating the screen (22). S290P was introduced into TSP-S238N-PETase (TSP-S238N-S290P-PETase or TSPNP-PETase) and purified enzyme displayed another 1.5 °C increase in thermostability relative to TSP-S238N-PETase with slight reduction in maximum productivity on 0.25”-diameter PET films (Figs. 1*D* and S3); the mutation was carried forward for further study due to the benefits to thermostability and purification. The locations of the three identified mutations are shown on the crystal structure of *Is*PETase in Figure 1*E* (24). To determine the kinetic impacts of the isolated mutations, the inhibition effect was further investigated to develop a comprehensive biochemical model for mutation analysis.

### *Is*PETase likely experienced crowded conditions on the surface of PET

**Figure 2:**
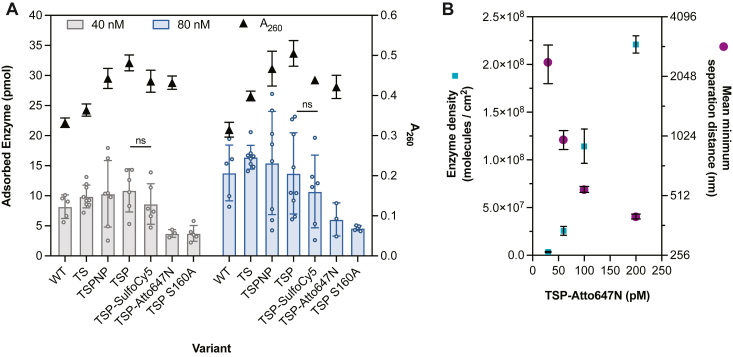
**Adsorption of *Is*PETase to amorphous PET films.***A*, solution depletion experiments were performed to determine amount of adsorbed enzyme molecules on the surface of 0.25”-diameter amorphous PET films (Goodfellow). Enzyme concentrations of 40 nM (*graybars*) and 80 nM (*bluebars*) were incubated with or without the film for 1.5 h at 30 °C, and fluorescence of proteins was used to measure supernatant enzyme concentration. *Black triangles* show the corresponding A_260_ product reading for each sample after 1.5 h. Difference between TSP-PETase and TSP-SulfoCy5 were insignificant for both enzyme concentrations based on unpaired two-tailed student *t* test (*p* > 0.1). Bars and error bars show mean and SD (*n* ≥ 3 biological replicates shown in *circles*). *B*, TIRF measurements of enzyme surface density and mean minimum separation distance between molecule centers on the surface of spun-coated PET thin films ([PET] = 5000 cm^2^/L) incubated with 0 to 200 pM of TSP-Atto647N. The mean and SD of the number of particles identified from eight equal quadrants (two locations on the PET film per enzyme concentration) are shown in *blue squares*. The mean and SD of the mean minimum separation distance from the eight quadrants are shown in *purple circles*. PET, poly(ethylene terephthalate).

Surface crowding has been previously proposed as the cause of the inhibition effect, where crowded environments led to decreased enzyme activity similar to effects of macromolecular crowding (9, 11, 17). To explore enzyme density on PET films during catalysis, purified *Is*PETase variants were incubated with 0.25”-diameter PET films for 1.5 h at 30 °C and the concentration of residual *Is*PETase in solution was used to infer adsorption (Fig. S4, *A*–*D*) (8). Intrinsic protein fluorescence allowed detection down to 10 nM of enzyme, but degradation products were found to artificially reduce fluorescence signal (Fig. S4, *E*–*G*). Thus, sample measurements were corrected according to its estimated MHET concentration, which is the major product (Fig. S4*H*). To avoid product interference and cross-validate the MHET-corrected measurements, two fluorescently-tagged (Atto647N and SulfoCy5) TSP-PETase variants and inactive TSP-S160A-PETase were also assayed. However, the addition of fluorescent tags may alter adsorption behavior, and the inactive variant cannot productively bind PET and may have reduced adsorption. With these caveats, average adsorption levels between 4 to 11 pmol at 40 nM of total enzyme and 5 to 16 pmol at 80 nM of total enzyme were measured for all variants (Fig. 2*A*). Considering 4 pmol of adsorbed enzyme on a completely smooth PET surface, it equated to 5 nm separation distance between evenly distributed particle centers or 2.5 nm mean minimum distance between randomly distributed particle centers based on Monte Carlo simulations. However, the surface of PET is not completely smooth on the nanometer scale, and therefore the separation distances between molecules were likely larger (27). Intermolecular interactions between enzymes as well as varying PET substrate accessibility across the surface can cause nonrandom distribution of molecules, which will also affect separation distance.

The enzyme density and distribution on the surface of PET were further explored using total internal reflection fluorescence microscopy (TIRF) with TSP-Atto647N. Though TSP-Atto647N showed the lowest adsorption in solution depletion experiments, Atto647N was chosen due to higher resistance to photobleaching and to obtain a lower-bound density estimate (molecules within 214 nm also could not be distinguished) (28). TSP-Atto647N (∼98% tagged) at concentrations of 0 to 500 pM were added to a PET-coated glass slide ([PET] = 5000 cm^2^/L) and imaged under a TIRF microscope (Fig. S5). Individual molecules were indistinguishable by 500 pM, while the mean minimum separation distance between enzyme centers was measured to be 400 nm at 200 pM (Figs. 2*B* and S5*B*). The distribution of enzyme particles on the spun-coated PET surface appeared to be random across all enzyme concentrations assayed (Fig. S5*B*). The onset of observable inhibition effects is reported to occur around 80 to 120 nM of total *Is*PETase with 630 cm^2^/L PET films, which is delayed to higher enzyme concentrations with increasing substrate concentrations (2, 9). If a linear increase in surface density (consistent with adsorption profile in Fig. S4*C*) was assumed to estimate separation distance at 75 nM enzyme and 5000 cm^2^/L PET, the simulated mean minimum separation distance of randomly distributed particle centers was found to be ∼20 nm (Fig. S6). Compared to a separation distance of 10 nm for crowding conditions induced by BSA and Ficoll 70 solutions in literature, the solution depletion and TIRF experiments together supported that *Is*PETase was likely experiencing crowded surface conditions over the enzyme-substrate concentration combinations where inhibition effects were observed. Finally, the adsorption experiments suggested that the amount of free enzyme in the reaction was <90% of the total enzyme concentration and enzyme excess cannot be assumed in kinetic analyses at the lower enzyme concentrations.

### Molecular dynamics simulations suggest that crowding reduces frequency of productive catalytic site conformations

**Figure 3:**
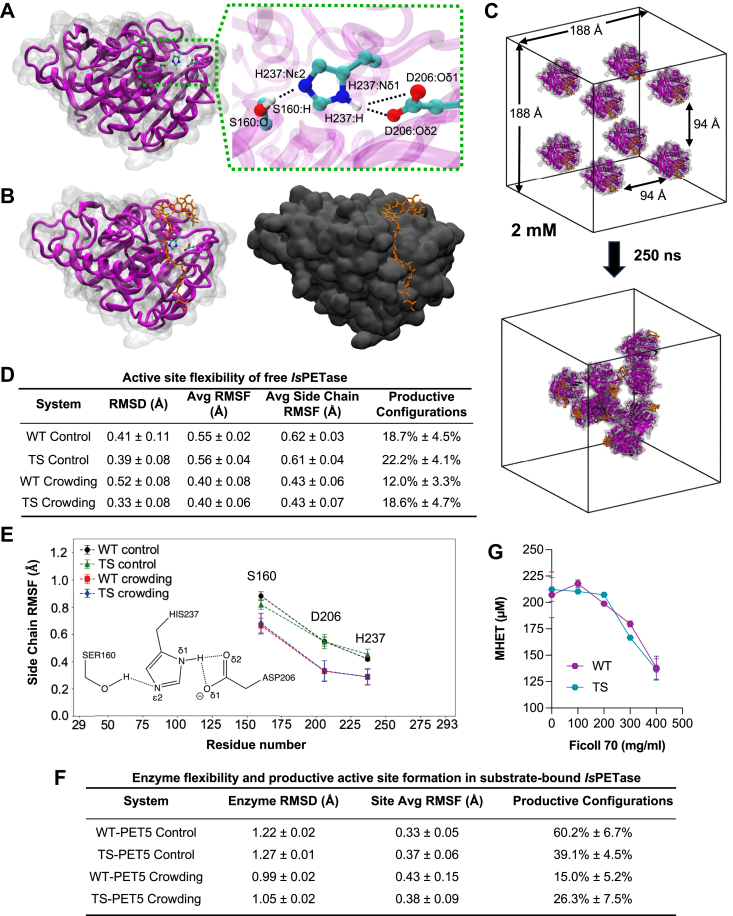
**Effects of crowding on the molecular dynamics of *Is*PETase.***A*, overall structure of WT *Is*PETase (PDB ID: 6EQE) with the catalytic triad (S160, D206, and H237) shown in ball-and-stick model (24). Key atom groups participating in hydrogen bond coupling for active site stabilization are labeled in close-up. *B*, MD model of substrate-bound WT *Is*PETase (WT-PET5) with the PET oligomer (PET5) substrate docked to the substrate-binding pocket in an energy-minimized configuration. *C*, initial and final configurations of substrate-bound WT *Is*PETase MD systems at the simulated crowding concentration of 2 mM; water molecules are not shown. *D*, summary of catalytic triad residue stability, flexibility, and observation of productive active site conformations for free *Is*PETase MD systems. Equilibrium active site RMSD, RMSF, side chain RMSF, and productive configuration percentage were assessed upon system convergence between 200–250 ns of simulation time. 99% confidence intervals are reported. *E*, residue side chain RMSF of the catalytic triad for free *Is*PETase MD systems. *F*, summary of overall enzyme stability, active site flexibility, and observation of productive active site conformations for substrate-bound *Is*PETase MD systems. Equilibrium enzyme backbone RMSD, active site RMSF, and productive configuration percentage were assessed upon system convergence between 200 to 250 ns of simulation time. Productive configurations are characterized by catalytic triad hydrogen bond coupling (S160-H237 & D206-H237) as illustrated in (*A*). 99% confidence intervals are reported. *G*, hydrolysis of BHET into MHET after 1 h at 30 °C by 40 nM of WT and TS-PETase in 0 to 400 mg/ml Ficoll 70. All data has been baseline corrected with BHET incubated in corresponding buffer without enzyme for 1 h at 30 °C. Each point represents the mean and error bars show SD (*n* = 3 biological replicates). BHET, bis(2-hydroxyethyl) terephthalate; MHET, mono-2-hydroxyethyl terephthalate; PET, poly(ethylene terephthalate).

To gain insights into macromolecular crowding effects on enzyme conformation and activity, we performed all-atom MD simulations of both free and PET-pentamer–bound WT and TS-PETase. The simulations were performed at an enzyme crowding concentration of 2 mM in solution (∼10 nm separation distance) to be representative of higher enzyme concentrations expected on a crowded PET surface. We compared our results against respective control simulations of a single uncrowded *Is*PETase variant in both the free and pentamer-bound state (Fig. 3, *A*–*C*). All MD analyses were performed over the last 50 ns of the converged simulation trajectory for both crowding and control simulations, at which time we observed that each system had achieved equilibrium. This was indicated by convergence of the RMSD in enzyme conformation and by complete monomer aggregation in crowding simulations. For uncrowded control simulations, results and error bars were reported as averages across three independent replica MD simulations. For crowding simulations, results and error bars were reported as averages across each of the eight enzymes in the simulation. The simulations demonstrated that crowding led to reduced overall enzyme flexibility and active site residue flexibility in free enzymes, while hindering the formation of productive conformations in substrate-bound enzymes (Fig. 3, *D* and *F*).

Under crowding conditions, it was observed that both WT and TS-PETase underwent aggregation through nonspecific attractive interactions, rendering them less mobile (Fig. 3*C*). Thus, the average flexibility of all eight enzymes in the system was reduced when compared to that of a single enzyme from the control simulation, which can be attributed to enhanced protein–protein surface interactions stabilizing enzyme conformations. The formation of protein-protein interfaces was stochastically driven and not related to specific residue interactions. An increase in the overall stability of individual enzymes can also be rationalized alongside inhibition behavior and longer enzyme life-times under the crowding hypothesis.

The *Is*PETase active site is composed of the S160, D206, and H237 catalytic triad, which is responsible for the hydrolysis of the ester linkages in PET. No significant change to the overall active site flexibility based on active site residue backbone RMSD was observed between control and crowding conditions for both WT (0.41 Å *versus* 0.52 Å) and TS (0.39 Å *versus* 0.33 Å) variants (Figs. 3*D* and S7*C*). To more comprehensively capture active site dynamics in the free enzyme, the flexibility of each catalytic triad residue was quantified using RMSF. The average RMSF of the three active site residues and their individual side chains across all free enzyme MD systems were drastically reduced under crowding conditions for both variants (Fig. 3, *D* and *E*). Notably, the changes in RMSF from control to crowding simulations of the isolated catalytic triad side chains were larger than for the entire residues (Figs. 3*E* and S7*D*), indicating that side chain mobility was the primary contributor to overall active site flexibility in the free enzymes. The importance of side chain dynamics was further explored in simulations of substrate-bound *Is*PETase variants.

We examined the effect of crowding on the frequency of productive active site formation in substrate-bound enzyme systems. Upon substrate binding, the catalytic triad residues were responsible for forming productive active site conformations through hydrogen bond coupling between S160-H237 and D206-H237 (29). Hydrogen bonding of S160 OH with N*ϵ*2-H of H237 can be coupled to H237 N*δ*1-H with either O*δ*1 or O*δ*2 of D206 (Fig. 3*A*), promoting D206-H237 charge stabilization and S160 deprotonation to carry out nucleophilic attacks against PET ester linkages for hydrolysis (Fig. 3*B*) (30). Thus, the probability of productive active site formation was quantified based on the frequency of simultaneous coupling of both hydrogen bond types in our MD simulations (3.5 Å donor-acceptor distance and 30° angle cutoffs). Under crowding conditions, we found that the probabilities of the substrate-bound enzyme in forming productive active sites were greatly reduced for both WT (60.2% to 15.0%) and TS-PETase (39.1% to 26.3%) (Figs. 3*F* and S7, *E*–*G*). Additionally, considerably lower probabilities of productive conformations were observed for uncrowded free enzyme compared to uncrowded substrate-bound enzyme for WT (18.7% *versus* 60.2%) and TS-PETase (22.2% *versus* 39.1%), while similar comparisons under crowding conditions indicated no significant differences between free and substrate-bound enzymes (Fig. 3, *D* and *F*).

To experimentally evaluate the MD simulation results, crowding conditions were simulated *in vitro* with 100 to 400 mg/ml of Ficoll 70, and the rate of hydrolysis of BHET into MHET by 40 nM of WT and TS-PETase were assayed. Ficoll 70 is a polar sugar polymer that has been shown to minimally interact with proteins and often adopts a spherical shape of approximately 5 nm in diameter, which is similar in size to *Is*PETase (20, 31). WT and TS-PETase activity was unchanged up to 200 mg/ml Ficoll 70, which suggested that the enzyme was not inhibited by Ficoll 70 through protein–polymer interactions (Fig. 3*G*). Both variants displayed similar decrease in activity between 200 to 400 mg/ml Ficoll 70 (Fig. 3*G*). The decreased BHET conversion observed in Ficoll 70 crowding experiments agree with the MD simulations, both suggesting that crowding will lead to decreased activity of *Is*PETase.

### An SC model for the analysis of *Is*PETase activity

Based on the adsorption data and molecular dynamics simulations, we propose a catalytic model that describes, through macromolecular crowding, the inhibition effect registered at high *Is*PETase concentrations. Since adsorption is likely much faster than catalysis, which is consistent with surface dynamics studies of PHL7 and LCC PETases, the two steps were modeled here as separate subsequent processes (32). Free enzymes *E* adsorb onto free substrate sites *N* and form adsorbed enzymes *EN*:

(1) $$E\;+\;N\;\overset{k_{a}}{\underset{k_{d}}{⇌}}\;EN$$

where *k*_*a*_ is the adsorption kinetic constant and *k*_*d*_ is the desorption kinetic constant.

The total concentration in the system of enzyme *E*_*T*_ and of substrate sites *N*_*T*_ are, respectively, given by:

(2) $$\left[E_{T}\right]=\left[E\right]\;+\;\left[EN\right]$$

(3) $$\left[N_{T}\right]=\left[N\right]\;+\;\left[EN\right].$$

Since *N*_*T*_ cannot be directly measured, we introduced the parameter Γ, representing the number of sites per unit surface of the substrate:

(4) $$\left[N_{T}\right]=\Gamma \left[N_{0}\right]$$

where *N*_0_ is the substrate surface concentration in the system (8). The parameter Γ depends on the enzyme variant and substrate type, as different mutants will have different abilities to access the substrate sites on the surface and different substrates types have different substrate sites (8). For example, PET chains are preferentially arranged in *gauche* conformation on the surface of amorphous Goodfellow PET films at 30 °C and very few are in *trans* conformation, but a mutation to *Is*PETase will cause the active site to prefer *trans* conformation chains, thus decreasing the number of available substrate sites (lowered Γ) on that PET substrate (30, 33). Under the assumption that adsorption is much faster than catalysis, a quasi-steady-state assumption was postulated for *EN* (32):

(5) $$\frac{d\left[EN\right]}{dt}=k_{a}\left[E\right]\left[N\right]-k_{d}\left[EN\right]=0.$$

Solving Eqs. 2, 3, and 5 for [*EN*],

(6) $$\left[EN\right]=\frac{1}{2}\;\left[\Gamma \left[N_{0}\right]\;\;+\;\;\left[E_{T}\right]\;\;+\;\;\frac{1}{K_{a}}\;-\;\sqrt{{\left(\left[E_{T}\right]\;\;+\;\;\Gamma \left[N_{0\;}\right]\;\;+\;\;\frac{1}{K_{a}}\right)}^{2}-\;4\left[E_{T}\right]\Gamma \left[N_{0\;}\right]}\;\right]$$

where *K*_*a*_ = *k*_*a*_*/k*_*d*_ is the adsorption equilibrium constant. Equation 6 allowed the direct computation of [*EN* ] from *E*_*T*_ and *N*_0_, which were known experimental quantities.

Adsorbed enzymes *EN* are present on the substrate surface in either an uncrowded configuration *EN*_*uc*_, performing catalysis with kinetic constant *k*_*cat,uc*_ or in a crowded configuration *EN*_*c*_, with lower catalytic rate constant *k*_*cat*,c_:

(7) $${EN}_{uc}\;\;+\;\;C_{n}\;\overset{k_{cat\text{,}\;uc}}{\to }\;{EN}_{\mathrm{uc}}\;+\;C_{n-1}\;+\;P$$

(8) $${EN}_{c}\;+\;C_{n}\;\overset{k_{cat\text{,}\;c}}{\to }\;{EN}_{c}\;+\;C_{n-1}\;+\;P$$

(9) $$\left[EN\right]=\left[{EN}_{\mathrm{uc}}\right]\;+\;\left[{EN}_{c}\right]$$

where *C*_*n*_ is a polymer (ex: PET) chain of length *n*, *C*_*n−*1_ is a polymer chain of length *n*–1, and *P* is a soluble degradation product. In practice, the adsorbed enzymes would have a distribution of catalytic kinetic constants based on the local crowding conditions. However, since it was not possible to measure the distribution of the catalytic rate across the adsorbed enzyme population, we here lumped all enzymes that were affected by crowding into *EN*_*c*_, which performed catalysis with kinetic constant *k*_*cat,c*_. The Supplementary Information further discusses this approximation.

From Eqs. 7 and 8, the overall polymer degradation rate is

(10) $$\frac{d\left[P\right]}{dt}=k_{cat,uc}\left[{EN}_{uc}\right]\;+\;k_{cat,c}\left[{EN}_{c}\right].$$

Assuming that the transition from uncrowded to crowded configurations (and vice versa) is much faster than catalysis, an equilibrium is quickly established between *EN*_*uc*_ and *EN*_*c*_:

(11) $${EN}_{uc}\;\overset{k_{c}}{\underset{k_{uc}}{⇌}}\;{EN}_{c}$$

(12) $$\frac{d\left[{EN}_{uc}\right]}{dt}=-\;\frac{d\left[{EN}_{c}\right]}{dt}=r_{uc}-\;r_{c}=0$$

where *k*_*c*_ (*k*_*uc*_) is the kinetic constant for the transition from uncrowded (crowded) to crowded (uncrowded) configuration and *r*_*c*_ (*r*_*uc*_) is the rate of transition from uncrowded (crowded) to crowded (uncrowded) enzyme configuration. The crowding and uncrowding reactions reported in Eq. 11 summarize a set of reactions that can lead to the transition from an uncrowded to a crowded configuration (and vice versa), as detailed in the Supplementary Information. Rates *r*_*c*_ and *r*_*uc*_ were computed, respectively, as (derivation in the Supplementary Information):

(13) $$r_{c\;}=k_{c}\left[{EN}_{\mathrm{uc}}\right]\left[EN\right]$$

(14) $$r_{\mathrm{uc}}=k_{\mathrm{uc}}\left[{EN}_{c}\right](\left[N_{T}\right]-\left[EN\right]).$$

Solving the system of Eqs. 9, 12 to 14 for [*EN*_*c*_] and [*EN*_*uc*_]:

(15) $$\left[{EN}_{uc}\right]=\left[EN\right]\frac{1\;-\;\theta }{K_{c}\theta \;\;+\;1-\theta }$$

(16) $$\left[{EN}_{c}\right]=\left[EN\right]\frac{K_{c}\theta }{K_{c}\theta \;\;+\;\;1\;-\;\theta }$$

where *K*_*c*_ = *k*_*c*_*/k*_*uc*_ is the crowding equilibrium parameter, and the site coverage *θ* is

(17) $$\theta =\frac{\left[ΕΝ\right]}{{[Ν}_{T}]}=\frac{\left[ΕΝ\right]}{\Gamma \left[{Ν}_{0}\right]}.$$

The polymer degradation rate (Equation 10) becomes

(18) $$\frac{d\left[P\right]}{dt}=k_{cat,uc}\frac{1-\;\theta \;\;+\;\;cK_{c}\theta }{{Κ}_{c}\;\theta \;+\;1\;-\;\theta }\;\left[ΕΝ\right]$$

where *c* = *k*_*cat,c*_*/k*_*cat,uc*_ represents the ratio between the catalytic rate constants of crowded and uncrowded enzyme configurations. Equation 18 can be reformulated as

(19) $$\frac{d\left[P\right]}{dt}=k_{app}\left[EN\right]$$

where the apparent catalytic kinetic constant *k*_*app*_ represents the average catalytic rate per adsorbed enzyme (*i.e.* productivity), which varies as a function of *θ* due to crowding according to

(20) $$k_{app}=k_{cat,uc}\frac{1-\theta \;+\;cK_{c}\theta }{{Κ}_{c}\theta \;+\;1-\theta }.$$

In the extreme scenarios of *θ* = {0, 1}, *k*_*app*_ becomes:

(21) $$k_{app}=\left\{ \begin{array}{c} {\;k}_{cat,uc},\;\text{for}\;\theta =0,\\ {\;k}_{cat,c},\;\text{for}\;\theta =1, \end{array}\right.$$

showing that *k*_*cat,uc*_ represents the maximum catalytic rate per adsorbed enzyme, corresponding to a site coverage *θ* = 0, while *k*_*cat,c*_ represents the minimum catalytic rate per enzyme, which is achieved at *θ* = 1 where the detrimental effect of crowding on the catalytic rate per enzyme reaches its maximum. Equation 20 clarifies the physical meaning of *K*_*c*_, which represents how quickly the decay of the catalytic rate per enzyme from *k*_*cat,uc*_ to *k*_*cat,c*_ occurs with increasing site coverage *θ* due to crowding.

The biochemical model of Equations 6, 17 and 18, together with the fitting parameters *K*_*a*_, *K*_*c*_, Γ, *k*_*cat,uc*_, and *k*_*cat,c*_ represents a closed form solution that, given the inputs *E*_*T*_ and *N*_0_, can predict the catalytic rate for a given mutant. We herein refer to this model as the SC model.

### Kinetic analysis of variants with the SC model

**Figure 4:**
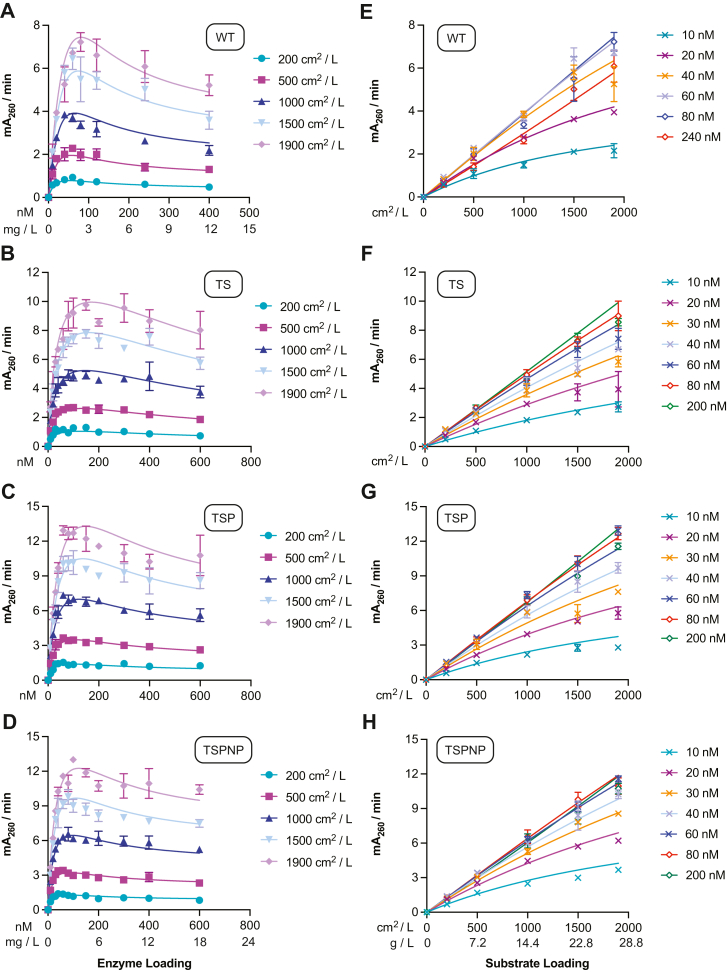
**Initial rates of *Is*PETase variants over 10 to 600 nM of enzyme and 200 to 1900 cm**^**2**^**/L (7.2–28.8 g/L) PET loading at 30°C.** All points represent the mean of three biological replicates and error bars show SD. Each replicate represents the rate of product accumulation between 30 to 210 min of the enzyme reaction with amorphous PET film. *A*–*D*, fit of SC model to initial rate *versus* enzyme concentration profiles. *E*–*H*, fitted SC model to same kinetic data plotted as initial rate *versus* substrate concentration for representative enzyme concentrations. PET, poly(ethylene terephthalate); SC, surface crowding.

We next used the SC model established by Equations 6, 17 and 18 to study the adsorption and catalytic behaviors of WT, TS-PETase, and the two evolved TS-PETase variants (TSP and TSPNP). The details of the parameter estimation approach are provided in the Experimental Methods section. Herein, we will refer to catalytic rate as the rate per enzyme molecule, while productivity is used to refer to the observed macroscopic product formation rate. The kinetic model appropriately captured the steady-state productivity of the *Is*PETases over a range of enzyme (10–600 nM) and PET (200–1900 cm^2^/L) loadings (Fig. 4) as measured using the bulk absorbance method (the relative rates were validated with HPLC upon reaction completion) (Figs. S8 and S9) (2). Notably all four *Is*PETases displayed crowding behavior at 30 °C. At low enzyme concentrations, productivity increased with increasing enzyme loading as more enzymes bound to PET. However, as the enzyme loading increased further, productivity slowed as the enzymes became crowded on the PET surface. The kinetic model also captured the steady-state adsorbed PETase concentrations (Equation 6), which began to plateau at higher enzyme loadings (Fig. S10). The estimated parameter values at 30 °C are summarized in Table 1.

**Table 1:** Fitted parameters from the SC kinetic model (30 °C)

Parameter	Value				
	WT	TS	TSP	TSPNP	Units
*K*_*a*_	0.0290	0.00196	0.00579	0.00847	nM^–^^1^
Γ	0.0291	0.141	0.0859	0.0701	nmol cm^–2^
*K*_*c*_	0.890	20.4	6.71	6.89	–
*k*_*cat, uc*_	0.445	1.11	0.932	1.01	mA_260_ min^–1^ nM^–1^
*k*_*cat, c*_	0.0588	0.00441	0.0386	0.0555	mA_260_ min ^–1^ nM^–1^

See Table S1 for 95% confidence intervals.

**Figure 5:**
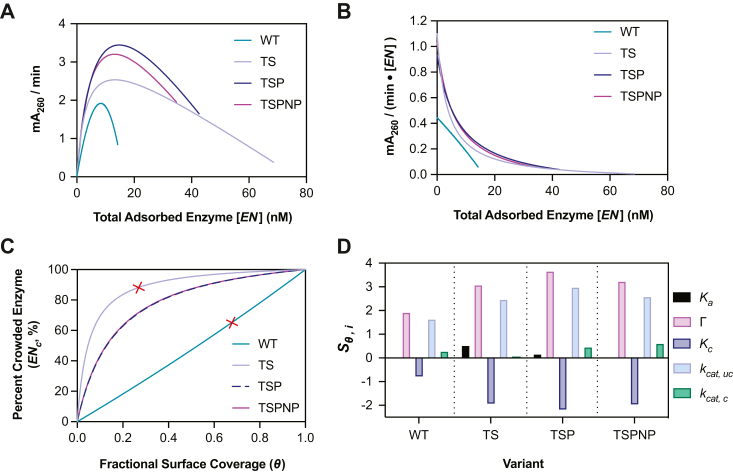
**Model-based analysis of catalytic activity of *Is*PETase variants.***A*, model prediction of catalytic rate *versus* adsorbed enzyme concentration. *B*, model prediction of average catalytic rate (productivity) per enzyme *versus* adsorbed enzyme concentration. *C*, percentage of enzymes in crowded configuration *versus* surface coverage *θ*. *Red* ‘×’ indicates the surface coverage at which inhibition effect is observed macroscopically for WT and TS-PETase. *D*, sensitivity of catalytic rate to the model parameters (Equation 22) for each variant, evaluated at the enzyme concentration *E*_*T*_ corresponding to the maximum productivity in Figure 4. In (*A* and *B*), each variant has a different maximum value of adsorbed enzyme concentration, related to Γ (Eq. 4). In all simulations, [*N*_0_] = 500 cm^2^/L.

TSP- and TSPNP-PETase achieved the highest productivity, followed by TS-PETase and WT, on both a total enzyme loading (Fig. 4) and predicted adsorbed enzyme basis (Fig. 5, *A* and *B*). All TS-PETase–based variants demonstrated higher catalytic rates in uncrowded conditions *k*_*cat,uc*_ and a larger Γ than the WT enzyme, which contributed to their higher productivity (Table 1). Interestingly, TS-PETase had lower productivity than the TSP and TSPNP mutants despite having comparable *k*_*cat,uc*_ and a larger Γ (Table 1). This phenomenon suggested that introduction of T116P decreased TS-PETase susceptibility to crowding at 30 °C, in both kinetics of transition to crowded configurations and *k*_*cat,c*_ (TSP-PETase presented smaller *K*_*c*_ and larger *k*_*cat,c*_, respectively) (Table 1). The S238N and S290P mutations had minimal impact on all kinetic parameters, thus their main contribution is to the thermostability of the enzyme. The model predictions of the specific catalytic rate per enzyme as a function of bound enzyme (Fig. 5*B*) and the concentration of enzymes in crowded configurations with respect to site coverage (Fig. 5*C*) further supported that adsorbed WT and TS-PETase were the least and the most affected by crowding, respectively. This was mainly due to WT having the lowest Γ and TS-PETase having the highest Γ based on model fits to adsorption data, which dictated the extent of SC conditions experienced by each variant (Table 1, Fig. S10).

To further characterize the changes in the kinetic parameters and the associated mutations that could improve enzyme productivity, we computed $s_{\theta_{i}}$, the sensitivity of productivity with respect to the model parameters:

(22) $$s_{\theta_{i}}=\theta_{i}\;\frac{\partial }{\partial \theta_{i}}\;{\left(\frac{d\left[P\right]}{dt}\right)}_{\theta_{j}\neq \theta_{\iota },{\;E}_{T},{\;N}_{0}}.$$

Sensitivities were computed at the enzyme concentration *E*_*T*_ at maximum productivity for *N*_*0*_ = 500 cm^2^/L. For all variants, Γ was the parameter most correlated with maximum productivity, followed by *k*_*cat,uc*_ and *K*_*c*_. While Γ and *k*_*cat,uc*_ were positively correlated with productivity, *K*_*c*_ showed a negative correlation, as expected (Fig. 5*D*). The parameters *k*_*cat,c*_ and *K*_*a*_ had a minor effect on maximum productivity.

### Crowding behavior may be masked at elevated reaction temperatures

**Figure 6:**
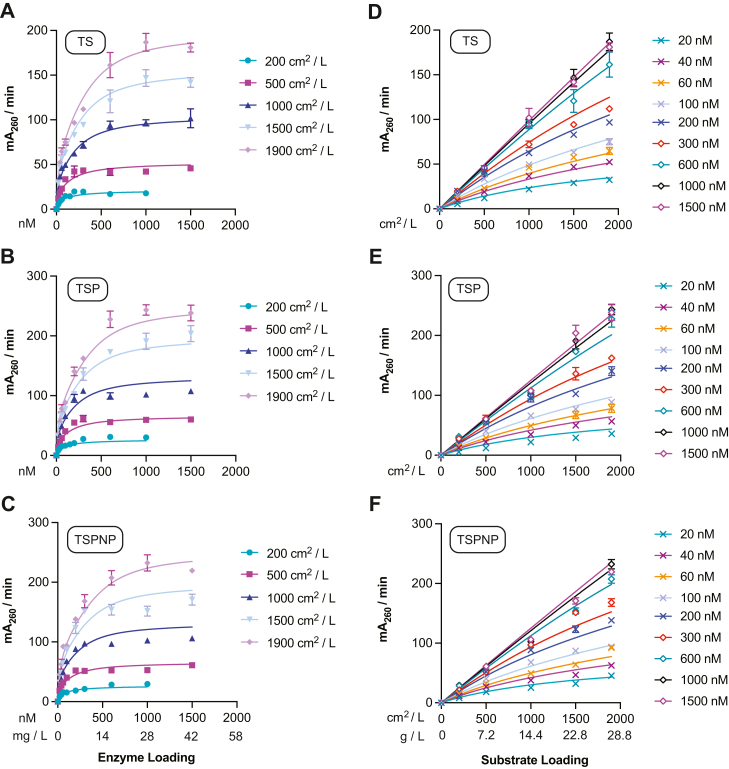
**Initial rates of *Is*PETase variants over 20 to 1500 nM of enzyme and 200 to 1900 cm**^**2**^**/L (7.2–28.8 g/L) PET loading at 55°C.** All points represent the mean initial rate of three biological replicates and error bars show SD. Initial rate was determined based on product formation between 15 to 105 min of the enzyme reaction with PET film. *A*–C, fit of SC model to initial rate *versus* enzyme concentration. *D*–F, fitted SC model to same kinetic data plotted as initial rate *versus* substrate concentration for each enzyme concentration. PET, poly(ethylene terephthalate); SC, surface crowding.

We used the SC model to study the effects of higher temperature on the adsorption and catalytic behaviors of the three TS-PETase variants. The kinetic model appropriately captured the steady-state productivity of the variants over 20 to 1500 nM enzyme and same PET loadings (200–1900 cm^2^/L) at 55 °C (Figs. 6 and S11). Similar to the 30 °C conditions, TSP- and TSPNP-PETase achieved higher productivity than TS-PETase. That said, the overall productivity for all conditions and enzymes increased by roughly an order of magnitude compared to 30 °C (Fig. 4), driven by a combination of more enzyme binding sites (Γ) and faster uncrowded and crowded catalytic rate constants (*k*_*cat,uc*_, *k*_*cat,c*_; Table 2). In contrast to 30 °C, however, no enzymes displayed the characteristic inhibition effect attributable to enzyme crowding. As a result, TSP-PETase produced 2.4 mM of combined TPA and MHET after 105 min at a substrate loading of 28.8 g/L (1900 cm^2^/L) and 1000 nM enzyme (Fig. S12). To investigate the lack of observed inhibition, we assessed the 55 °C data using the SC and ^*inv*^MM models (Figs. 6 and S13). Bayesian information criterion analysis (Table S3) indicated that the additional parameters in the SC model provided a significant fitting improvement over the ^*inv*^MM model, suggesting that crowding was still needed to appropriately explain the kinetic behavior of the enzymes at high temperatures (34). The steady-state TSP-PETase adsorption data plateaued at a higher concentration at 55 °C (Fig. S14) than at 30 °C (Fig. S10*C*) and was appropriately described by the model.

**Table 2:** Fitted parameters from the SC kinetic model (55 °C)

Parameter	Value			
	TS	TSP	TSPNP	Units
*K*_*a*_	0.00793	0.00832	0.00869	nM^–^^1^
Γ	0.214	0.228	0.269	nmol cm^–2^
*K*_*c*_	21.2	22.8	22.5	–
*k*_*cat, uc*_	3.77	4.71	4.23	mA_260_ min^–1^ nM^–1^
*k*_*cat, c*_	0.489	0.582	0.492	mA_260_ min ^–1^ nM^–1^

See Table S2 for 95% confidence intervals.

## Discussion

Macromolecular crowding is a known phenomenon that impacts protein dynamics in many ways from protein stability and conformation to catalytic activity (16, 35). The effects of crowding vary dramatically between crowding agents and the biochemistry of the protein itself. Here, we propose a model where a dilute enzyme solution phase can result in a crowded surface environment upon protein adsorption, leading to macromolecular crowding that can affect enzyme activity. Based on our MD simulations, one possible explanation is that sufficient space for the necessary active site conformational changes during hydrolysis is required for optimal enzyme performance, which is consistent with reduced catalytic efficiency under crowding. For free enzymes, active site flexibility would allow easier transition to conformations that can readily bind to the substrate (36). Interestingly, active site flexibility was inherently reduced upon substrate binding, resulting in minimal differences in active site flexibility between crowded and uncrowded states (Fig. 3*F*). However, the frequency of productive conformations sampled by substrate-bound enzymes was greatly reduced under crowding. Per residue side chain RMSF differences (Fig. 3*E*) and atomic separation distance shifts between S160 OH and H237 N*ϵ*2-H groups (Fig. S7*E*) suggested that S160 mobility drove hydrogen bonding between S160 and H237 and limited productive site formation (Fig. S7, *F* and *G*). Thus, we can attribute the decreased frequency of productive enzyme configurations under crowding to sterically induced stabilization of unfavorable S160 positions. Notably, substrate-bound enzymes sampled more productive active site configurations compared to free enzymes, suggesting that substrate binding to the active site itself had an effect in stabilizing favorable S160 positions. The overall stability of substrate-bound enzyme was also affected by macromolecular crowding as evident from the reduced protein backbone RMSD (Fig. 3*F*). It was previously observed that increased enzyme concentrations led to decreased productivity, but the enzyme concentrations displaying inhibition behavior also sustained the reaction over longer periods of time (2). This observation is consistent with the crowding hypothesis, which can result in more compact conformations of *Is*PETase that may have led to increased enzyme stability, resulting in longer life times of *Is*PETase at higher concentrations.

Using enzyme crowding as the basis for the inhibition effect, we developed a two-state kinetic model of enzymatic catalysis of solid PET, which is referred to as the SC model. The model captured the expected shift in optimal enzyme concentration with increasing substrate concentration as previously observed by Avilan *et al.* (2023) (Fig. 4) (9). Prior knowledge of a variant’s adsorption to PET is required to estimate an upper bound for Γ and fit *K_a_*. Direct measurement of substrate site density would result in an even more robust analysis, but it is extremely difficult to determine and cannot be reliably fitted using ^*inv*^MM and ^*conv*^MM as previously done for other systems due to the inhibition effect (8). Therefore, the adsorption capacity was used as the upper bound for fitting the number of substrate sites for each variant and the distinction between adsorption (Γ_*ads*_) and substrate site density (Γ_*kin*_) was not made. However, an enzyme may be able to adsorb to nonspecific sites, resulting in higher adsorption capacity than substrate site density, which may obscure analysis if the proportions are very different between variants for relative comparisons (8, 37).

Interestingly, the current SC model Equation 20 reduces down to the Mukai 1993 rate equation if *K_c_* = 1, *k*_*cat,c*_ = 0, along with the inclusion of a substrate term, which shows that the models are related (13). The SC model is a more comprehensive model that removes several assumptions made in the Mukai model, such as enzyme excess, zero activity at full site coverage (likely true for two-domain but not necessarily for single-domain depolymerases), and that mutations had no effect on enzyme susceptibility to crowding (13). Thus, the proposed SC model provides more detailed insights into effects of mutations and is likely more broadly applicable to all solid-substrate depolymerases. Formally assessing the SC model’s applicability to multidomain enzymes would require collecting analogous datasets to those presented in Figures 4 and 6 and can be the focus of a future study.

**Figure 7:**
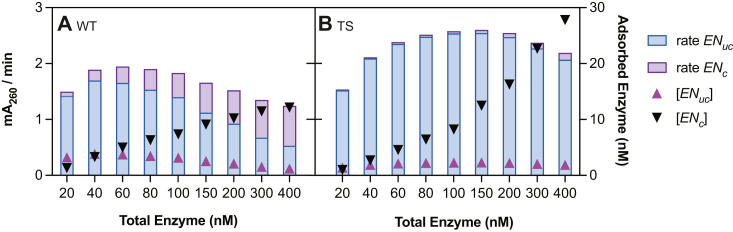
**Predicted impacts of crowding on WT and TS-PETase at 30 °C.** Contribution of uncrowded (*EN*_*uc*_) and crowded (*EN*_*c*_) enzymes to the observed productivity (mA_260_/min) based on the SC model for (*A*) WT and (*B*) TS-PETase. *Bars* show rate contributions (*left* axis) and *triangles* show absolute concentrations of each species predicted by the SC model (*right* axis). Predictions were made using [*N*_0_] = 500 cm^2^/L. SC, surface crowding.

The SC model showed that the true catalytic capacity will never be experimentally observed in macroscopic experiments due to crowding, which may also explain the discrepancy between single-molecule (uncrowded) and macroscopic rate measurements for cellulases (11). Furthermore, it suggested that fitting with MM models will underestimate intrinsic catalytic rate (^*conv*^MM, ^*inv*^MM, and general MM fits of increasing portion of data can be found in Figs. S15 and S16, Tables S4 and S5). The SC model also revealed that WT *k*_*cat,c*_ was the least impacted by crowding, partly due to its lowered adsorption capacity compared to the TS variants (fewer enzymes on the surface to induce similar levels of crowded conformations) (Fig. 5, *B* and *C*). This result could appear counter intuitive to observations from kinetic data where WT displayed a 28% productivity inhibition at 400 nM total enzyme while TS-PETase only had 12% inhibition at 400 nM, highlighting the importance of coupling adsorption equilibrium experiments to kinetic analysis for complete data interpretation. Adsorption experiments showed that WT approached full site coverage faster due to its low Γ and high *K*_*a*_; thus the model predicted a more pronounced macroscopic effect of crowding as loss of uncrowded complexes were not compensated by an increase in total adsorbed enzyme, while the opposite was predicted for TS-PETase (Fig. 7). Therefore, at a given enzyme loading, WT productivity was the most sensitive to crowding at a macroscopic scale, despite individual enzymes maintaining the highest catalytic activity in crowded configurations. On the contrary, TS-PETase productivity was most resistant to crowding, as the high crowding effects were compensated with a higher adsorption capacity (Fig. 5*C*).

The SC model predicts that the most desirable mutations for improving enzymatic PET degradation will (i) increase Γ and (ii) the *k*_*cat,uc*_, while (iii) decreasing *K*_*c*_. This is consistent with reports where fusion of a polymer-binding module to PET-hydrolases increased enzyme productivity (increased Γ) (38, 39, 40, 41, 42, 43). However, it was also found that a few hydrophobic modules, which greatly increased the productivity of some thermostable PET-hydrolases, sometimes resulted in lowered *Is*PETase productivity (38, 41, 42). This could be due to a large increase in Γ that resulted in a large population of crowded WT-fusion enzymes, which led to lower productivity due to susceptibility of the WT-fusion to crowding effects (reduced *k*_*cat,c*_ and likely increased *K*_*c*_). On the contrary, inhibition effects are less commonly observed in thermostable PET-hydrolases, suggesting that increasing their binding capacity is more likely to lead to an increase in observed productivity (9).

In conclusion, we present a kinetic model based on the hypothesis of macromolecular crowding on the solid substrate surface during enzymatic depolymerization. The phenomenon is applicable to many ambient-temperature solid-substrate depolymerases and may also be relevant for high-temperature reactions, which are very important for future bio-economies such as biorecycling and biovalorization of food wastes (9, 11, 13). The implications of the model will likely have large impacts on enzyme engineering for improved yields, as decreasing crowding tendency alone can boost productivity of the enzymes. Analysis of kinetic data using the model will allow identification of the types of mutations that provide resistance to crowding, which can then be probed using protein dynamics methods such as NMR to better understand the structure-function relationships of crowding behavior. Finally, fundamental knowledge of structure-crowding relationships will allow predictions of beneficial mutations and improve the efficiency of a large number of critical bioprocesses.

## Experimental methods

### Cloning and plasmid constructs

All plasmids used in this study were generated using the pET21a+ backbone (2). Briefly, *Is*PETase (residues 28–290) was codon optimized for expression in *E. coli* and cloned into pET21a+ with a C-terminal His-tag using PCR with Phusion polymerase (New England BioLabs) and primers listed in Table S6. The PCR fragments were joined together using NEBuilder HiFi DNA Assembly Mix (New England BioLabs). All constructs contained a His_6_-tag except for plasmids used for directed evolution, which had a C-terminal His_8_-tag. The C-terminal His_8_-tag was introduced into the pET21a-*Is*PETase plasmid using pET21a His_8_ F/R primers. Plasmids were propagated using *E. coli* TOP10F′ (New England BioLabs) in LB+100 μg/ml carbenicillen medium (herein referred to as LB+Carb). Site-directed mutagenesis was performed as previously described to introduce T116P, S238N, and S290P into the TS-PETase background (Table S6) (2). The LPETGG Sortase recognition tag was introduced in front of the C-terminal His_6_-tag for Sortase-tagging of fluorescent peptides onto the C-terminus of TSP-PETase (primers: pET21a Srt F and *Is*PETase Srt R, Table S6).

### Cloning of directed evolution libraries

*In silico* mutant libraries of TS-PETase were synthesized by Twist Biosciences and cloned into the pET21a+ plasmid. Round one–directed evolution library was generated using WT *Is*PETase as template and the following PCR conditions for a final reaction of 50 μl: 2 μl of 10 mM dGTP/dATP, 10 μl of 10 mM dCTP/dTTP, 10 μl 55 mM MgCl_2_, 5 μl 1 mM MnCl_2_, 3 μl of 10 μM *Is*PETase mut F/R, 2 fmol template DNA, 1 μl GoTaq Polymerase, and 33.6 μl water (44). The PCR was amplified 20 cycles. Round two–directed evolution library was generated using TSP-PETase as template and the following PCR conditions: 5 μl 10× GeneMorph II buffer, 100 ng template DNA, 2.5 ng/μl *Is*PETase mut F/R, 1 μl of 40 mM dNTP, 1 μl GeneMorph II polymerase (Agilent), and 37.5 μl water. The PCR was amplified 23 cycles to aim for 0 to 4.5 mutations/fragment. The pET21a+His_8_ backbone was amplified with Phusion polymerase and pET21a F/R. The PCR products were DpnI-digested for 6 h at 37 °C and gel purified (Zymo DNA Gel Purification Kit). Ligation was performed using NEBuilder HiFi DNA assembly mix at a 1:1 M ratio of insert to backbone (New England BioLabs) at 50 °C for 6 h. All primer sequences are listed in Table S6.

### *In silico*–directed evolution

A disulfide bridge at N233C-S282C was introduced into the crystal structure of *Is*PETase^S121E/D186H/R280A^ (PDB: 6IJ6) (7). The final structure was minimized to account for the effect of the additional mutations and protonated accordingly at pH 9 using Yasara software (http://www.yasara.org) and H++ webserver (http://newbiophysics.cs.vt.edu/H++/) to generate the TS-PETase structure for *in silico* evolution (45, 46). Several computational methodologies were employed to computationally evolve TS-PETase, each targeting distinct parts of the enzyme’s structure, namely distal positions and the active site (see Supplementary Information for detailed methods).

### Purification of *Is*PETase variants for biochemical analysis

pET21a+-*Is*PETase-His_6_ constructs were transformed into *E. coli* T7 Express using heat shock as described in Supplementary Information. Three to four transformants were grown overnight in 10 ml LB+Carb at 37 °C and 200 rpm. The overnight starter was inoculated 1:100 into 1L Terrific Broth+100 μg/ml carbenicillen (TB+Carb) and grown at 37 °C and 200 rpm for 3.5 h; then protein expression was induced with 1 mM isopropyl β-d-1-thiogalactopyranoside and grown overnight at 16 °C and 200 rpm. The culture was centrifuged at 5000 rpm for 10 min at 4 °C and medium removed. The cell pellet was resuspended to a final volume of 50 ml in 50 mM Hepes pH 7.2, 50 mM NaCl, 20 mM imidazole (resuspension buffer). 0.5 mg of DNaseI was added to the suspension and the cells were sonicated at 4 °C. The lysate was centrifuged for 30 min at 10 k rpm and 4 °C, and the supernatant was filtered through a 0.22 μm filter. The supernatant was incubated with 1 ml of Ni-NTA agarose resin (Thermo Fisher Scientific) for 2 h (overnight for WT) at 4 °C with shaking in a 50 ml falcon tube. The lysate was flowed through a gravity column, and the resin was washed five times with 10 ml of resuspension buffer. The elution buffer (50 mM Hepes pH 7.2, 50 mM NaCl, 300 mM imidazole) was flowed through the column until minimal signal was detected at A_280_. The elutions were incubated at room temperature for 2 h to precipitate contaminants (for TS-PETase variants only) and then pooled for size-exclusion chromatography. Five milliliters of pooled elution (maximum 50 mg of protein based on A_280_) was flowed through a PrepGrade S75 Hi-Load column (Cytiva) (running buffer: 25 mM Hepes pH 7.2, 50 mM NaCl) and fractions were collected in 2.5 ml intervals. All fraction peaks were collected and ran on SDS-PAGE gels, which were stained with Coomassie blue R250 (10% acetic acid, 40% methanol, 50% water); percent purity was assessed using the Fiji densitometry tool (47). The fractions with *Is*PETase that corresponded to the monomeric size of the protein were pooled and dialyzed into 50 mM glycine-NaOH pH 9, 50 mM NaCl overnight at 4 °C. S75 purification results in removal of all residual protein and nucleic acid contaminants. The amount of protein was determined using DC protein assay (Bio-Rad) in a 96-well microplate according to manufacturer directions.

### Differential scanning fluorimetry

Thermostabilities of purified enzymes were measured using differential scanning fluorimetry. Enzyme concentrations between 15 to 25 μM were mixed with SyproOrange (1:1000 dilution from stock) to a final volume of 10 μl in a 96-well qPCR plate. The fluorescence intensity was measured using a Bio-Rad CFX96 Real-Time PCR machine (FRET channel) from 10 to 95 °C with 5 s incubation steps for every 0.5 °C increment.

### Enzyme kinetic experiments

All bulk absorbance kinetic experiments were performed as previously described in 1.5 ml microfuge tubes (GeneMate) (2). Amorphous PET film substrates (Goodfellow USA, ES30-FM-000145) were cut to their respective surface areas: 2 × 5 mm, 5 × 5 mm, 10 × 5 mm, 15 × 5 mm, 19 × 5 mm. The 0.25”-diameter amorphous PET films were custom cut by Goodfellow (ES30-FM-000145). All PET films were washed once in 100% isopropanol, followed by three washes in 70% ethanol and air dried. Triplicate reactions were run for each substrate size and enzyme concentration with shaking at 200 rpm. All reactions were performed with the following reaction buffer: 50 mM glycine-NaOH pH 9, 50 mM NaCl, 10% v/v dimethyl sulfoxide (DMSO); the buffer was warmed to the reaction temperature prior to the experiment. A total volume of 1 ml of enzyme in reaction buffer was incubated with PET, and 1.25 μl of reaction supernatant was taken for measurement on the NanoDrop1000 (Thermo Fisher Scientific) at each time point (every 30 min for 30 °C, 15 min for 55 °C).

### Sortase A-tagging of TSP-PETase

TSP-PETase containing a C-terminal LPETGG-His_6_-tag was expressed in *E. coli* T7 Express and purified as described above. The following procedure was performed in the dark. GGGYK-C(Atto647N)-T(amide) peptide (Millipore Sigma) or GGGYK-K(SulfoCy5)-T(amide) peptide (21st Century Biochemicals) were used in the tagging reactions. Forty micrograms of Sortase A (SrtA) was incubated with 200 μl of Ni-NTA agarose slurry (Thermo Fisher Scientific) in 150 mM Hepes pH 7.2, 150 mM NaCl, 10 mM CaCl_2_ and loaded onto a 2 ml gravity column. Peptide (28 μM) and enzyme (20 μM) (or 7 and 5 μM for TIRF experiments, respectively) were mixed together to a final volume of 3 ml and flowed over the gravity column containing SrtA and flow-through was collected. The flow-through was flowed over the column five more times. The column was washed with 3 ml of 7 μM peptide and fraction collected. The flow-through was dialyzed into 500 ml of 50 mM glycine-NaOH pH 9, 50 mM NaCl three times at 4 °C using 10 kDa-cutoff dialysis tubing (ThermoFisher Scientific). The dialyzed sample was analyzed using fluorescence SDS-PAGE (Alexa 647 or Cy5 setting, Bio-Rad ChemiDoc MP) to ensure proper tagging and removal of peptide. Tagging percentage was calculated using A_646_ to determine concentration of fluorescent molecules and DC protein assay to measure total enzyme concentration.

### *Is*PETase adsorption to PET films

*Is*PETase at various concentrations was incubated with or without 0.25”-diameter amorphous PET films (Goodfellow USA, ES30-FM-000145) in 1 ml of reaction buffer in 1.5 ml microfuge tubes (GeneMate). The tubes were placed in a 30 °C incubator and shaken at 200 rpm for 1.5 h (45 min for 55 °C). Fluorescence was measured in three technical replicates for each sample: 250 μl/well of reaction supernatant was transferred to a UV-transparent flat-bottom black plate (Greiner Bio-One) and intrinsic protein fluorescence was measured (280/345 nm). Fluorescently tagged TSP-PETase was measured at 646/672 nm (SulfoCy5) and 646/675 nm (Atto647N). A_260_ was also measured to determine relative product concentrations in the supernatant. For intrinsic fluorescence measurements, MHET concentration was calculated based on the A_260_ value and the expected reduction in fluorescence was determined based on the MHET 280/345 nm fluorescence standard curve (Fig. S4*G*). The expected fluorescence loss was added onto the 280/345 nm fluorescence signal of the corresponding sample, and the corrected value was used to interpolate enzyme concentration using the protein fluorescence standard curve (10–1000 nM) for each variant. The raw fluorescence intensity of the reaction without PET was used to interpolate enzyme concentration using the same standard curves. The difference in enzyme concentration between the reaction with PET and without PET was used to calculate the amount of adsorbed enzyme onto the PET film.

### Spin-coating of PET thin films onto glass slides

Amorphous PET film (Goodfellow, ES30-FM-000145) was dissolved in 85% trifluoroacetic acid at 2% w/v and immediately used for spin-coating. 1”-diameter circular glass slides (170 μm thick, #1.5H) were cleaned with 70% ethanol and placed in the center of the spin-coater stage. Dynamic spin-coating was performed at 3 k rpm with 100 μl of PET solution for each glass slide for 60 s. A thin film of PET was found to form over the glass slide that can be peeled off. TSP-Atto647N was also spotted onto the spun-coated glass slides and product formation (A_260_) was detected, showing successful coating.

### TIRF microscopy

0 to 500 pM solution of TSP-Atto647N in 50 mM glycine-NaOH pH 9, 50 mM NaCl, 10% v/v DMSO was applied to a PET-coated glass slide. A sealed chamber using 0.5”-diameter O-ring and vacuum grease was constructed with 2 glass slides (one PET-coated and one uncoated) to prevent evaporation during sample imaging; each chamber contained 250 μl of sample. The sample was imaged in a single-molecule TIRF microscope with 638 nm excitation. The laser power was reduced to 0.9 to 1.0 mW at the objective to reduce PET background auto-fluorescence (32). See Supplementary Information for microscope details.

### TIRF data processing and analysis

Identification and extraction of individual fluorescence traces from camera images, as well as sorting of molecules were achieved using single-molecule platform for automated analysis (SPARTAN), a data analysis software tool created for sCMOS data sets (48). The SPARTAN software has been modified to disable automated background corrections. Molecule detection parameters were set to auto-detection sensitivity between 6 to 15 depending on image, and minimum distance between overlapping molecules was set to zero to detect all molecules on the surface; sensitivity was lowered to ensure sufficient molecule detection or increased to avoid background noise detection. SPARTAN produces a list of x-y coordinates for all detected molecules, which were used to count the number of molecules in each quadrant of the field of view (56 μm-diameter). This was done for each enzyme concentration at two different locations on the surface. Number of molecules detected in buffer controls were subtracted from total number of detected molecules in enzyme samples by randomly removing coordinates (total number of coordinates removed equaled number of molecules detected in blank) from the lists prior to separation distance determination. Using Fortran code, the distance between each coordinate and all other coordinates were calculated and the minimum separation distance was determined for each enzyme concentration and quadrant in the field of view. The result is a distribution of minimum distances for all of the points. The mean of the distribution is recorded as the mean minimum distance between points. The mean minimum distance from each of the eight quadrants for each enzyme concentration was used to determine SD.

### Molecular dynamics simulations of *Is*PETase variants with PET substrate and macromolecular crowding

The initial configuration for control enzyme monomer MD simulations was generated by centering the free or substrate-bound *Is*PETase variant in an orthorhombic box. A minimum distance of 3 nm was set between the enzyme and the edge of the box to prevent self-interaction across the periodic boundary conditions. Enzyme crowding simulations were set up by placing eight identical free *Is*PETase or substrate-bound *Is*PETase configurations in a 188 × 188 × 188 Å^3^ box spaced evenly apart. This results in approximately 94 Å between every enzyme pair’s center-of-masses corresponding to a 2 mM concentration. All systems were solvated with the TIP3P water model (49) and the total system charge was neutralized using chlorine ions. TS-PETase model was attained by performing *in silico* mutagenesis of the WT *Is*PETase crystal structure (24) (PDB ID: 6EQE) in PyMOL (50) and parametrized with the AMBER14SB force field (51). PET pentamer substrate model used in docking and MD simulations was parametrized using the General Amber Force Field 2 (GAFF2) (52). Molecular docking of the PET substrate to the *Is*PETase variants was performed in AutoDock Vina (53), with a chosen PET configuration that is consistent with previously reported docking experiments (6, 24). MD simulations were performed in GROMACS (54). Following energy minimization and equilibration steps, MD production simulations for all systems were performed for 250 ns in the NPT ensemble at 300K and 1 bar. Constant temperature and pressure were maintained by a velocity rescale thermostat (55) and a Parrinello-Rahman barostat (56), respectively. All simulations utilized a 2 fs time step with the LINCS (57) algorithm to constrain bonding between hydrogen and heavy atoms. Long-range electrostatic interactions were calculated using Particle Mesh Ewald summation with a 1.2 nm cutoff (58). From 250 ns MD simulations of the free and substrate-bound *Is*PETase variants under crowding and control conditions, protein structure equilibrium was observed in all systems after 200 ns so all analysis was performed over the final 50 ns of simulation trajectories. Simulation trajectories were post-processed in GROMACS and visualized with Visual Molecular Dynamics (59). RMSD and RMSF were performed with their respective built-in GROMACS functions. Hydrogen bonding calculation among enzyme catalytic triad residues (29) (S160, D206, and H237) was performed with 3.5 Å donor-acceptor distance and 30° angle cutoffs using the HBonds plugin tool in VMD. The occurrence of hydrogen bonds for the D206-H237 residue pair is counted for either O*δ*1 or O*δ*2 of D206 hydrogen bonding with N*δ*1-H of H237. Quantification of sampled productive active site conformations from hydrogen bonding analysis of catalytic triad residues was carried out similarly as outlined in the work of Guo *et al.* on *Is*PETase mutants (30).

### Macromolecular crowding with Ficoll 70

Ficoll 70 was dissolved in 50 mM glycine-NaOH pH 9, 50 mM NaCl, 10% v/v DMSO (reaction buffer) at a concentration of 400 mg/ml and subsequently diluted to 100, 200, and 300 mg/ml using reaction buffer. All buffers were centrifuged at 5000 rpm for 1 min to remove air bubbles. BHET, at a final concentration of 500 μM, was added to all buffer conditions and incubated with 40 nM of *Is*PETase (or no enzyme for blank controls) in 500 μl reactions for 1 h at 30 °C. Reactions were stopped 1:1 with 200 mM phosphate pH 2.5 buffer and filtered for HPLC analysis (See Supplementary Information for HPLC methods).

### Parameter estimation for kinetic model

The biochemical kinetic model established by Equations 6, 17 and 18 contains five fitting parameters (*K*_*a*_, *K*_*c*_, Γ, *k*_*cat,uc*_, and *k*_*cat,c*_). Parameters were fit in two steps for each of the studied enzymes. In the first step, adsorption parameters (*K*_*a*_ and Γ) were fit using maximum-likelihood estimation in the log space of the parameters (60).

(23) $$\hat{\theta }=\arg_{\theta }\;\min\;{\left(y-\tilde{y}\left(\theta \right)\right)}^{T}V_{\epsilon }^{-1}\left(y-\tilde{y}\left(\theta \right)\right)$$

where $\hat{\theta }$ is a vector of the estimated parameters, **y** is a vector containing the experimental observations, $\tilde{y}$ (***θ***) is a vector containing model predictions with parameter set ***θ***, and ***V***_***ϵ***_^*−*1^ is the measurement uncertainty covariance matrix. These adsorption parameters then served as priors for the second step of fitting, in which all five parameters (*K*_*a*_, *K*_*c*_, Γ, *k*_*cat,uc*_, and *k*_*cat,c*_) were fit using *maximum a posteriori* estimation in the log space of the parameters

(24) $$\hat{\theta }=\arg_{\theta }\;\min\;{\left(y-\tilde{y}\left(\theta \right)\right)}^{T}V_{\epsilon }^{\;-1}\left(y-\tilde{y}\left(\theta \right)\right)+{\left(\theta -\mu \right)}^{T}V_{\mu }^{\;-1}\left(\theta -\mu \right)$$

where ***μ*** is a vector of the parameter values predicted in the prior step and ***V***_***μ***_ is a matrix of the covariance of the parameters fit in the prior step. This formulation instills a penalty for adjusting the parameters fit in previous steps. The values for ***μ*** and ***V***_***μ***_ are set such that the parameters being fit for the first time in the second or third steps do not instill any cost.

Optimization and simulation were performed using MathWorks MATLAB software. Optimization was performed using a multi-start approach with an interior point algorithm and 2000 random initialization points for each enzyme. All parameters were scaled by log(***θ***) during optimization to improve the speed of searching the parameter space and to constrain parameter values to positive values.

## Data availability

All raw data are available upon request (enzhong@mit.edu).

## Supporting information

This article contains supporting information (61, 62, 63, 64, 65, 66, 67).

## Conflict of interest

The authors declare that they have no conflicts of interest with the contents of this article.
