# Supplementary material for: Analysis of Poly(ethylene terephthalate) degradation kinetics of evolved IsPETase variants using a surface crowding model
Source: J Biol Chem. 2024 Feb 22;300(3):105783. doi: 10.1016/j.jbc.2024.105783 (PMC10963241; doi:10.1016/j.jbc.2024.105783)
Supplement: Supporting Information [file mmc1.pdf]

Supplementary Information: Analysis of poly(ethylene terephthalate) degradation kinetics of evolved *Is*PETase variants using a Surface Crowding model

En Ze Linda Zhong-Johnson,[†] Ziyue Dong,[‡] Christopher T. Canova,[¶] Francesco Destro,[¶] Marina Cañellas,[§] Mikaila C. Hoffman,[‖] Jeanne Maréchal,[†,@] Timothy M. Johnson,[⊥] Gabriela S. Schlau-Cohen,[‖] Maria Fátima Lucas,[§] Richard D. Braatz,[¶] Kayla G. Sprenger,[‡] Christopher A. Voigt,[#] and Anthony J. Sinskey[*,†]

†*Department of Biology, Massachusetts Institute of Technology, Cambridge, MA, USA*

‡*Department of Chemical and Biological Engineering, University of Colorado, Boulder, CO, USA*

¶*Department of Chemical Engineering, Massachusetts Institute of Technology, Cambridge, MA, USA*

§*Zymvol Biomodeling SL, Barcelona, Spain*

‖*Department of Chemistry, Massachusetts Institute of Technology, Cambridge, MA, USA*

⊥*Plasma Science and Fusion Center, Massachusetts Institute of Technology, Cambridge, MA, USA*

#*Department of Biological Engineering, Massachusetts Institute of Technology, Cambridge, MA, USA*

@*AgroParisTech, 22 place de l'Agronomie, Palaiseau, France*

E-mail: asinskey@mit.edu

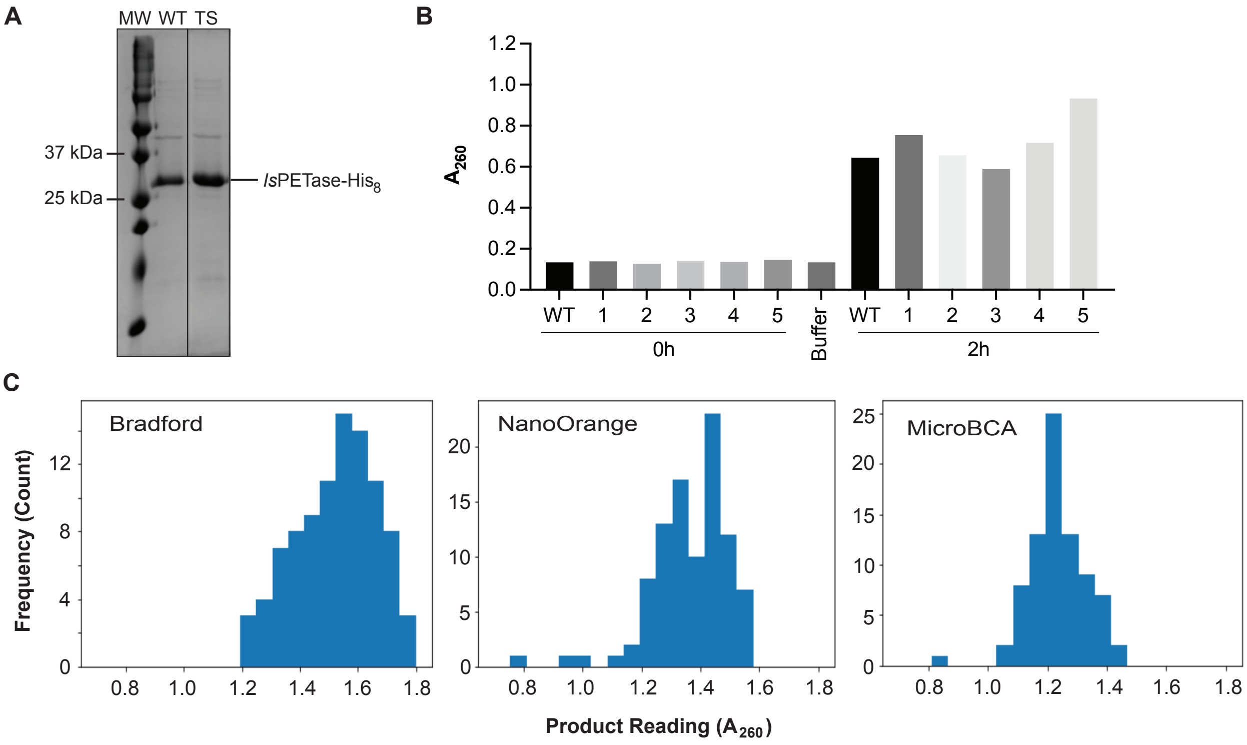

Figure S1. Directed evolution screen development. A) Coomassie blue-stained SDS-PAGE gels of high-throughput screen elutions of WT and TS-PETase using CelLytic Express (Millipore Sigma) and magnetic Ni-NTA resin (Genscript). Lanes were spliced together to omit irrelevant samples. MW = molecular weight ladder (Bio-Rad, Precision Plus Protein Dual Color). B) A_{260} readings in a 384-well plate of random variants 1-5 and WT high-throughput screen elutions normalized to 100 nM pre- (0h) and post-incubation (2h) with PET. C) Distribution of measured product accumulation (A_{260}) across a 96-well plate of wild-type *Is*PETase purified using the developed high-throughput purification strategy. Elutions were quantified with and normalized based on Bradford, NanoOrange (ThermoFisher), or MicroBCA (ThermoFisher) assay. Frequency is the number of wells in the plate within a range of A_{260} readings.

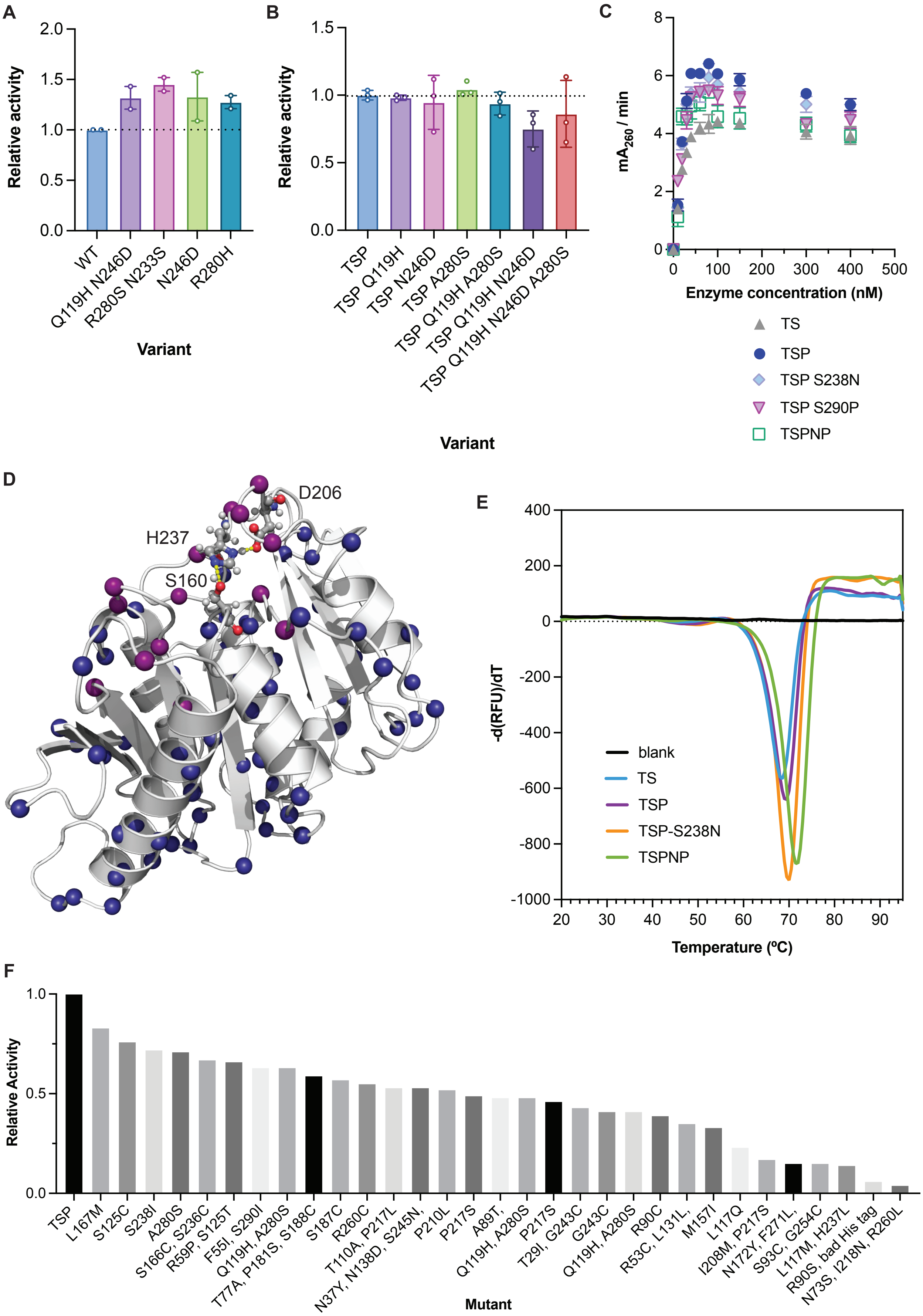

A
Relative activity
2.0
1.5
1.0
0.5
0.0
WT
Q119H N246D
R280S N233S
N246D
R280H
Variant
B
Relative activity
1.5
1.0
0.5
0.0
TSP
TSP Q119H
TSP N246D
TSP A280S
TSP Q119H A280S
TSP Q119H N246D
TSP Q119H N246D A280S
Variant
C
8
6
4
2
0
mA260 / min
0
100
200
300
400
500
Enzyme concentration (nM)
TS
TSP
TSP S238N
TSP S290P
TSPNP
D
D206
H237
S160
E
400
200
0
-200
-400
-600
-800
-1000
-d(RFU)/dT
20
30
40
50
60
70
80
90
Temperature (ºC)
blank
TS
TSP
TSP-S238N
TSPNP
F
Relative Activity
1.0
0.5
0.0
TSP
L167M
S125C
S238I
A280S
S166C, S236C
R59P, S125T
F55I, S290I
Q119H, A280S
T77A, P181S, S188C
S187C
R260C
T110A, P217L
N37Y, N138D, S245N,
P210L
P217S
A89T,
Q119H, A280S
P217S
T29I, G243C
G243C
Q119H, A280S
R90C
R53C, L131L,
M157I
L117Q
I208M, P217S
N172Y, F271L,
S93C, G254C
L117M, H237L
R90S, bad His tag
N73S, I218N, R260L
Mutant

Figure S2. Directed evolution results. A) Validation screen of round 1 library mutants. Activity relative to wild-type *Is*PETase (WT) is shown. Bars and error bars represent mean and range of biological duplicates (circles). B) Validation screen of round 1 library mutations of interest in the TSP-PETase background. Activity relative to TSP-PETase is shown. Bars and error bars show mean and standard deviation (n = 3 biological replicates are shown in circles). C) Initial product formation rate of purified variants identified from directed evolution on ¼"-diameter amorphous PET films at 30°C and 200 rpm. Points and error bars show mean and standard deviation (n = 3 biological replicates). D) Location of identified hotspots on TS-PETase for *in silico* directed evolution. Blue sites are distal sites, and purple sites are proximal sites to the catalytic tunnel. E) Melt curves of TS-PETase, TSP-PETase, TSP-S238N-PETase, and TSPNP-PETase in 50 mM glycine-NaOH pH 9, 50 mM NaCl (blank). Each melt curve is the mean of three biological replicates. F) Variants from round 2 library demonstrating decreased activity compared to TSP-PETase. All mutations are in TSP-PETase background.

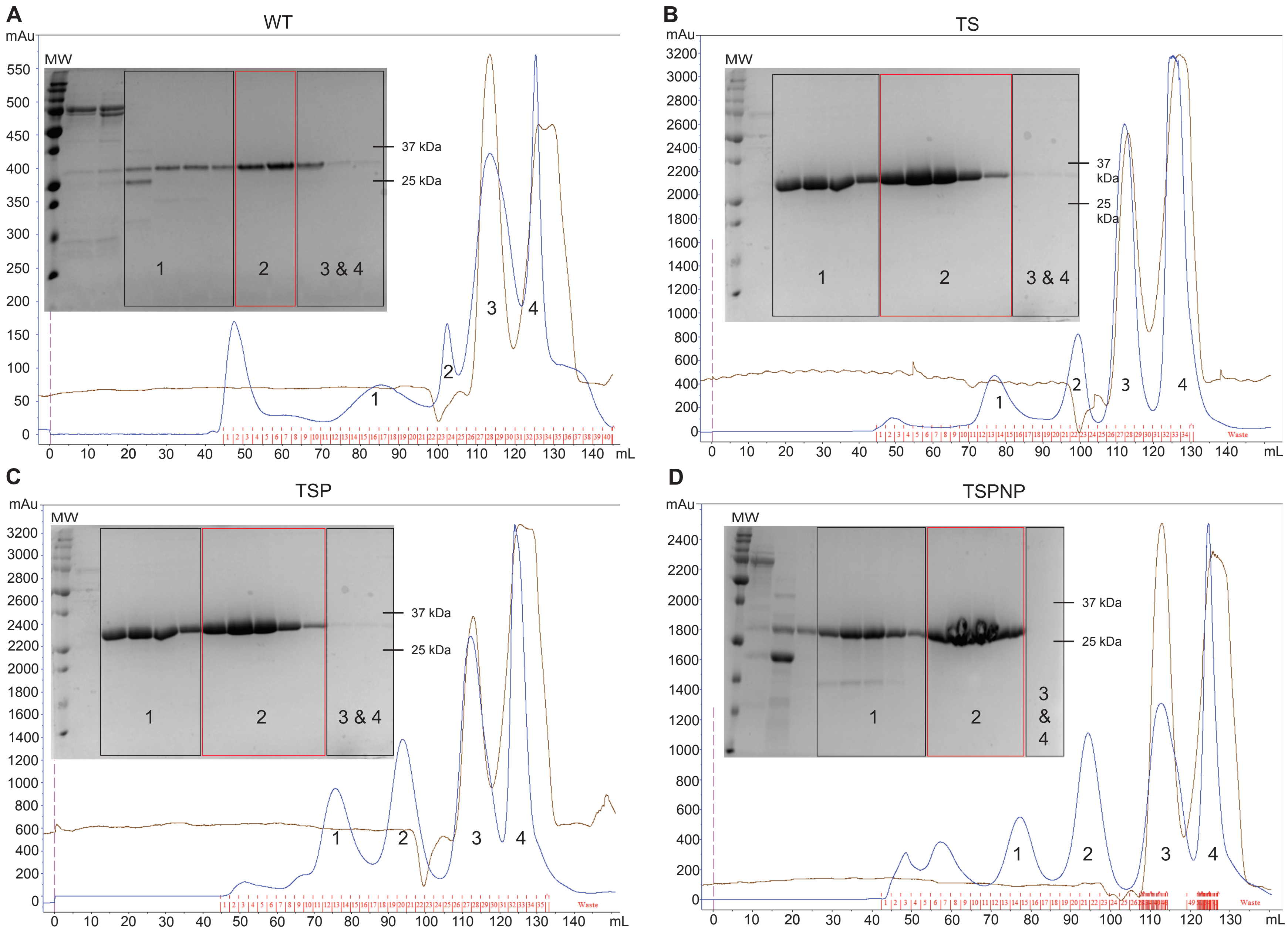
A
WT
mAu
MW
1
2
3 & 4
37 kDa
25 kDa
mL
B
TS
C
TSP
D
TSPNP
Waste

Figure S3. Coomassie blue-stained SDS-PAGE gels of S75 HiLoad size exclusion chromatography (SEC) fractions. This is the last step of protein purification from 1 L *E. coli* T7 Express cultures. 12 µL per fraction were loaded onto the gel. Each numbered box refers to the corresponding numbered peak on SEC traces. Samples within each red box were pooled for biochemical assays. All pooled samples show > 99% purity based on SDS-PAGE. Blue curves show A_{280} readings (reported as mAu on the y-axis) and brown curves show conductivity. Peaks 3 and 4 are likely small non-protein contaminants and imidazole present in the injected sample. MW = molecular weight ladder (Bio-Rad, Precision Plus Protein Dual Color).

[h!]

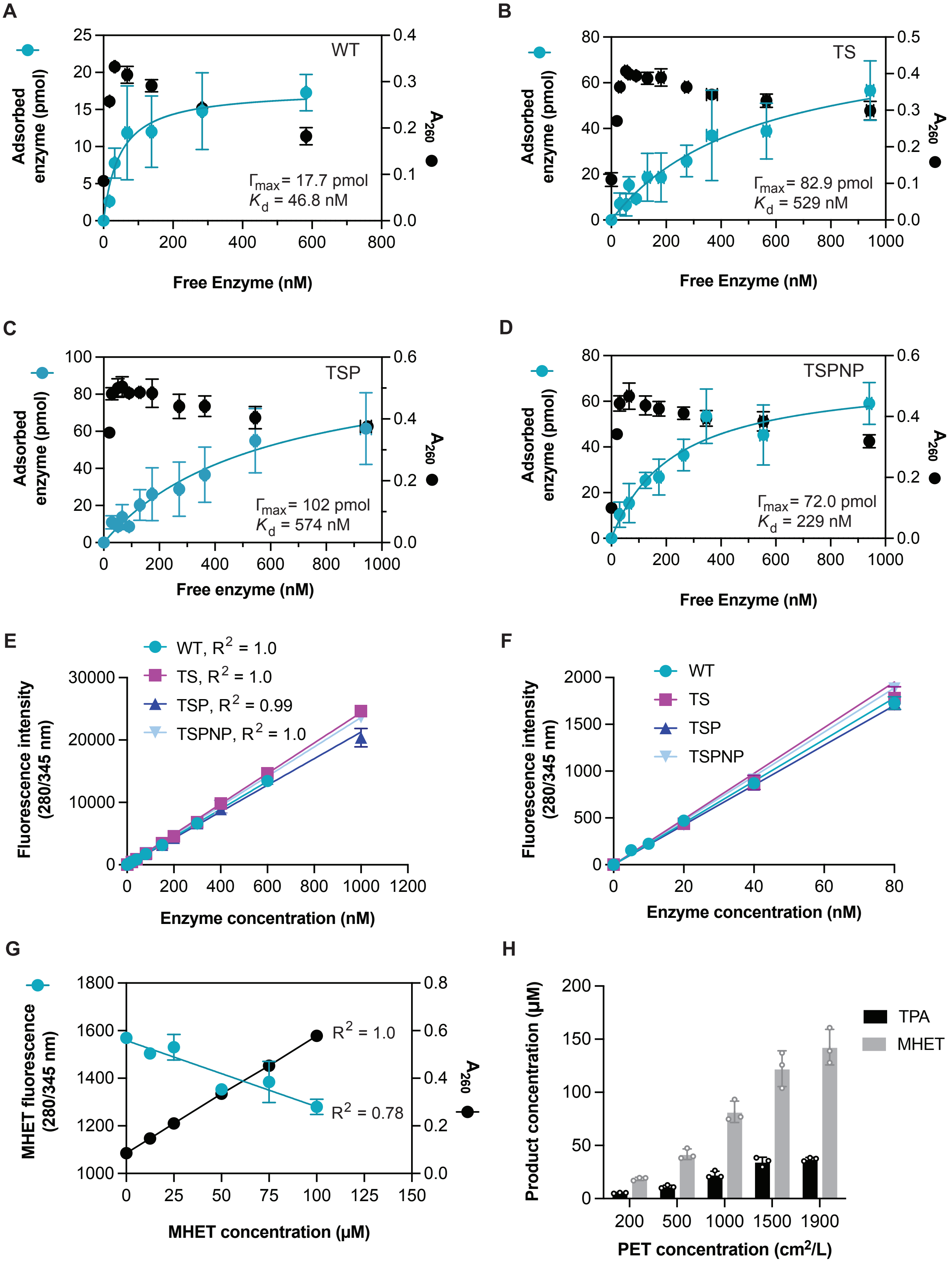
A
Adsorbed enzyme (pmol)
A260
WT
Γmax= 17.7 pmol
Kd = 46.8 nM
Free Enzyme (nM)
B
TS
Γmax= 82.9 pmol
Kd = 529 nM
Free Enzyme (nM)
C
TSP
Γmax= 102 pmol
Kd = 574 nM
Free enzyme (nM)
D
TSPNP
Γmax= 72.0 pmol
Kd = 229 nM
Free Enzyme (nM)
E
WT, R2 = 1.0
TS, R2 = 1.0
TSP, R2 = 0.99
TSPNP, R2 = 1.0
Fluorescence intensity (280/345 nm)
Enzyme concentration (nM)
F
WT
TS
TSP
TSPNP
Fluorescence intensity (280/345 nm)
Enzyme concentration (nM)
G
MHET fluorescence (280/345 nm)
R2 = 1.0
R2 = 0.78
A260
MHET concentration (μM)
H
Product concentration (μM)
TPA
MHET
PET concentration (cm2/L)

Figure S4. Adsorption of *Is*PETase to ¼"-diameter PET films. PET was incubated with enzyme for 1.5 hours at 30°C, and the amount adsorbed was determined based on the difference in supernatant enzyme concentration between no PET and PET samples. Mean and standard deviation of $n \geq 3$ biological replicates are plotted for A-D). Adsorption of A) WT, B) TS-PETase, C)TSP-PETase and D) TSPNP-PETase to PET film. All adsorption curves were fitted using the Langmuir adsoprtion model according to Badino *et al.* (2022) and best-fit values are shown for each variant (1). Caution should be taken in interpreting the values as the use of the Langmuir adsorption model is intended as a visual guide of the data. Interestingly, the K_d fitted for WT in this study is similar to K_d obtained by Badino *et al.* (22 ± 7 nM) on semi-crystalline PET powder. Supernatant enzyme concentration was calculated using intrinsic fluorescence at 280/345 nm. Standard curves of *Is*PETase 280/345 nm fluorescence between E) 5-1000 nM and F) 5-80 nM. G) MHET fluorescence at 280/345 nm and absorption at 260 nm. As PET products absorb light at 345 nm, the amount of product in each enzyme sample was estimated using A_{260} and fluoresence absorbed by the corresponding concentration of MHET was added to the corresponding sample fluorescence before determining supernatant enzyme concentration. Symbols and error bars show mean and standard deviation of three technical replicates for E-G). H) Distribution of MHET and TPA products at 40 nM of TSP-PETase as measured by HPLC after 3.5 hours of incubation at 30°C and 200 rpm. Bars and error bars show mean and standard deviation of three biological replicates (circles).

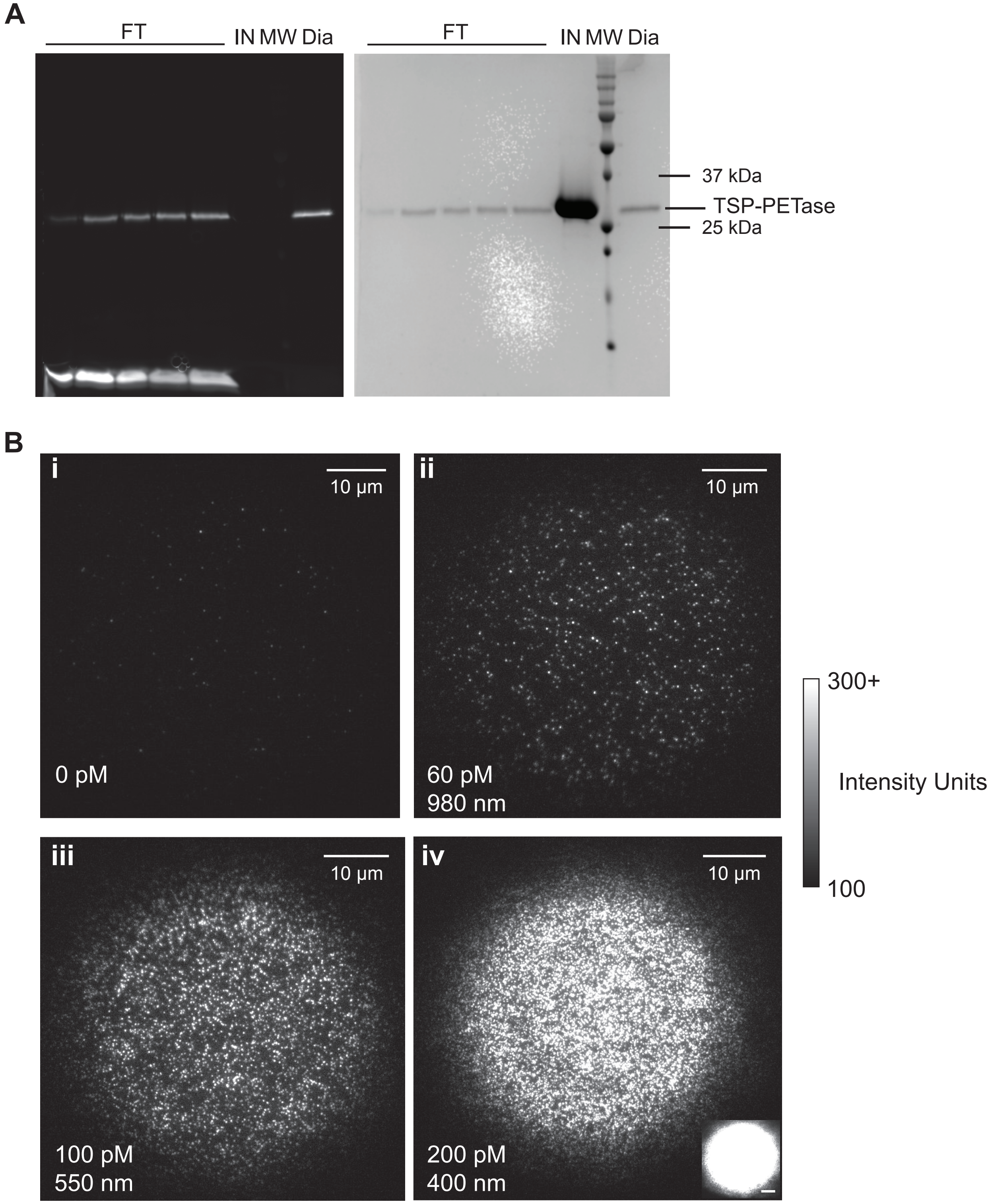

Figure S5. TIRF data. A) Sortase-tagging of TSP-PETase (C-terminal LPETGG-tag) with GGGYK-C(Atto647N)-T(amide) peptide. FT are flow through fractions from tagging reaction; IN is input protein; MW is the protein ladder (Bio-Rad, Precision Plus Protein Dual Color); Dia is dialyzed final FT used for experiments. Left: fluorescence image of gel using Alexa 647 setting on Bio-Rad ChemiDoc MP imager. Right: Coomassie Blue stain of the same gel. B) Representative TIRF images at each TSP-Atto647N concentration. The mean minimum separation distance for each enzyme concentration is shown in the bottom left corner. Panel iv) bottom right corner is image at 500 pM enzyme; small white bar in the bottom right corner shows 10 μm for the 500 pM image. All images were set to the same lower and upper bound intensities.

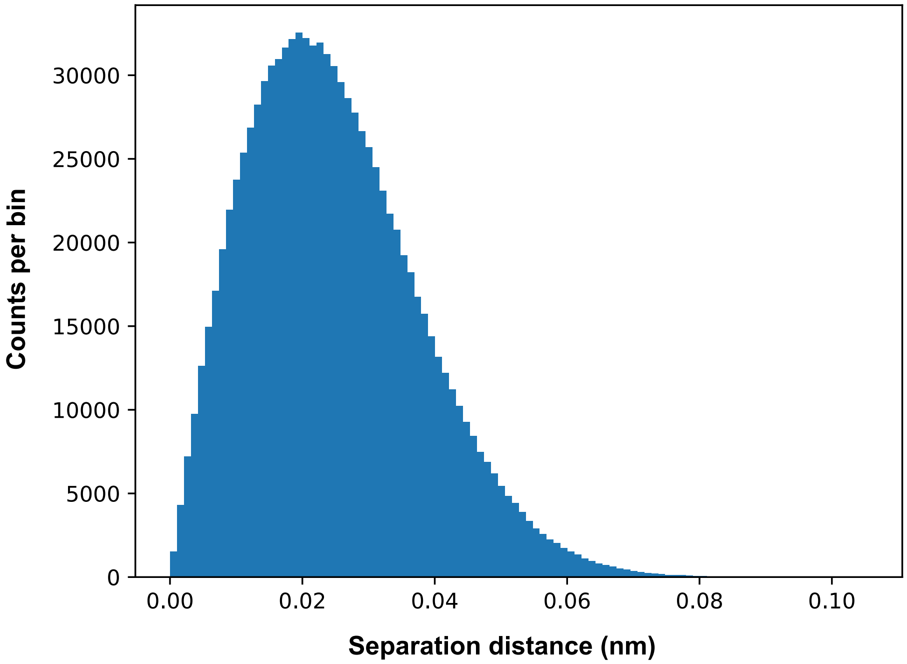

Figure S6. Monte Carlo simulation of 2.025 $\times 10^6$ particles on a 56 µm disk. This is the estimated number of enzyme molecules at 75 nM enzyme loading if the number of surface molecules increased linearly with respect to total enzyme concentration.

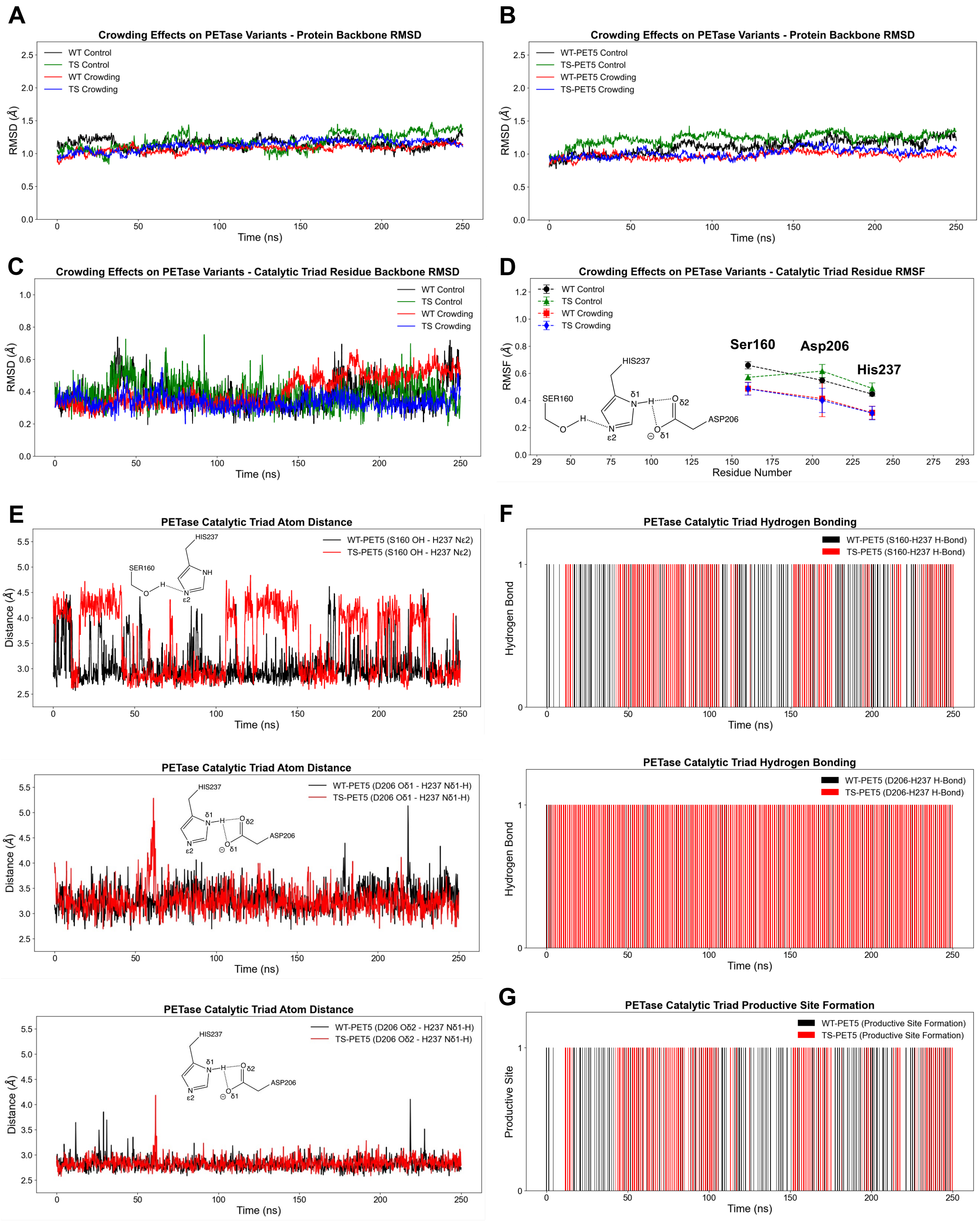

Figure S7. Protein backbone RMSD of the overall enzyme from MD simulations of A) free *Is*PETase and B) substrate-bound *Is*PETase. C) Residue backbone RMSD and D) residue RMSF of the catalytic triad from MD simulations of free *Is*PETase. E-G) Catalytic triad residue hydrogen bond coupling (S160 OH - H237 Nϵ2, D206 Oδ1 - H237 Nδ1, and D206 Oδ2 - H237 Nδ1) E) key atomic separation distances and F) hydrogen bonding occurrences. A vertical bar indicates the existence of F) hydrogen bonding between residue pairs and G) any resulting productive active site conformation from hydrogen bond coupling at the corresponding simulation time.

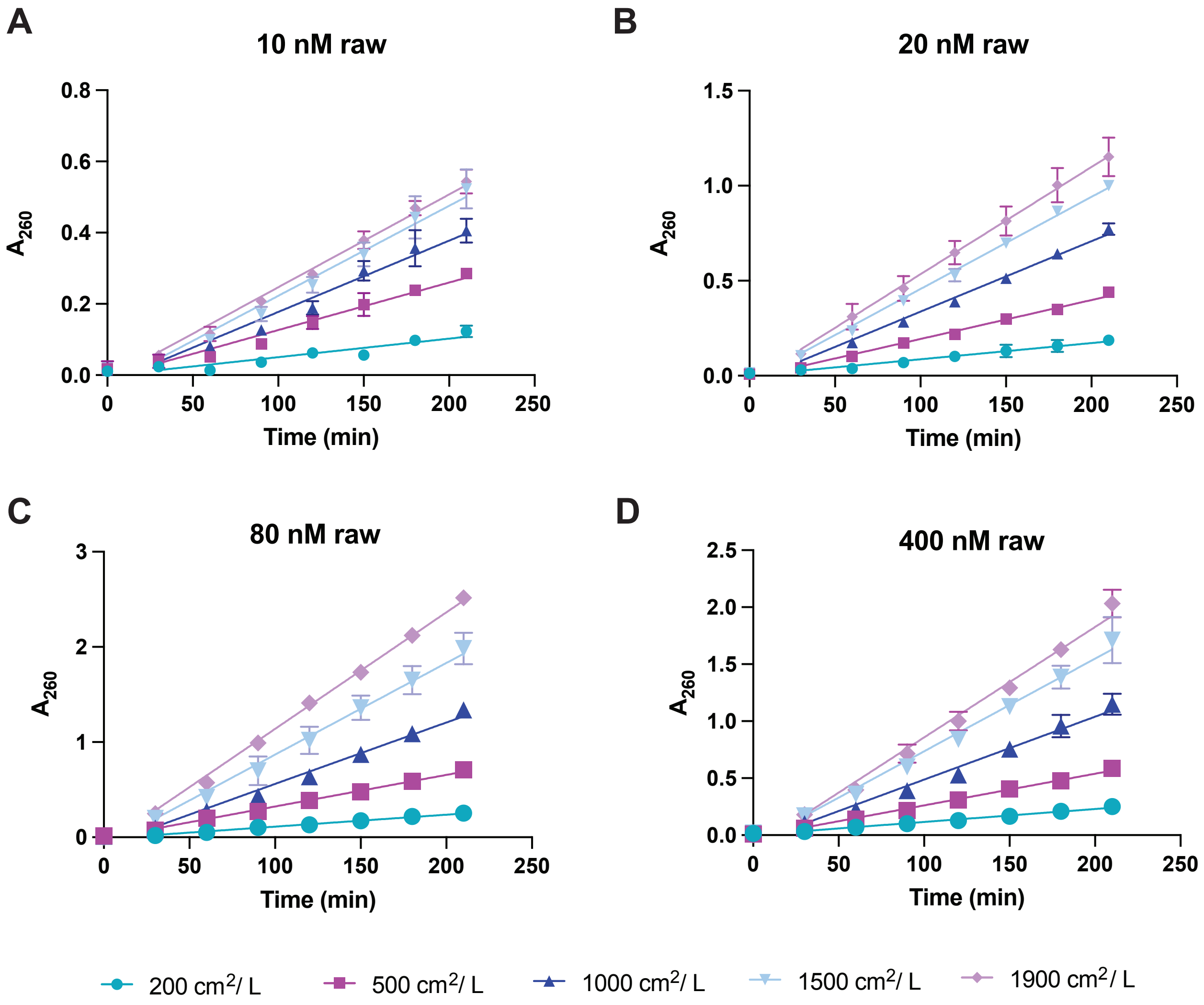

Figure S8. Raw initial rate data. Representative raw A_{260} values measured for TSP-PETase at 30°C within the first 3.5 hours for four different enzyme concentrations across 200-1900 cm^2/L PET loading. All initial rates are calculated based on the slope between 30 minutes and 3.5 hours. Mean and standard deviation are shown ($n = 3$ biological replicates).

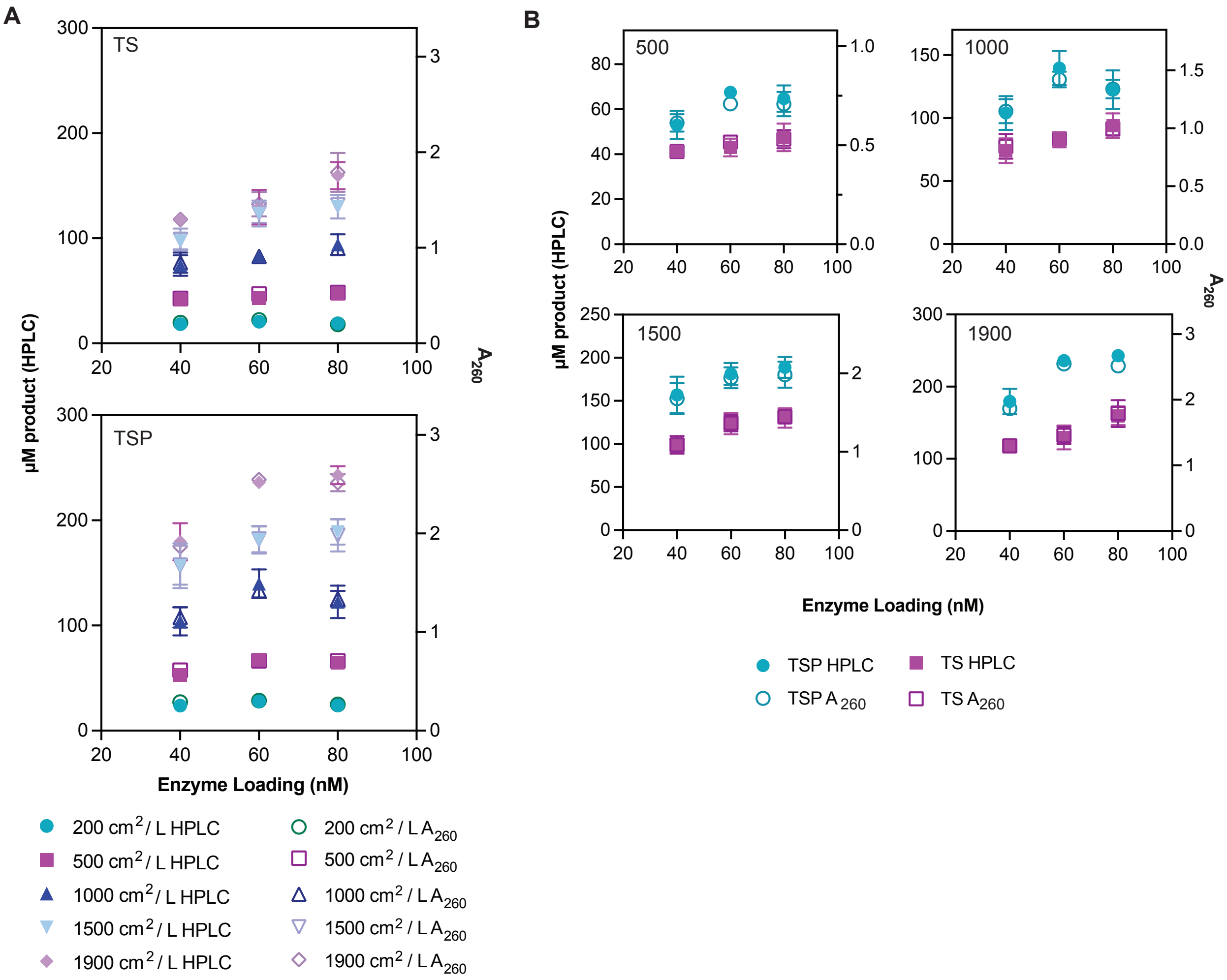

Figure S9. Cross validation of relative bulk absorbance product measurements at 30°C against HPLC. All HPLC product measurements are sums of TPA and MHET. A) HPLC vs. A_{260} measurements of product accumulation at 30°C and 3.5 hours for top: TS-PETase, bottom: TSP-PETase. B) Differences in product accumulation between TS-PETase and TSP-PETase at 30°C after 3.5 hours as measured via HPLC vs. A_{260}. Four representative substrate concentrations are shown: 500, 1000, 1500, 1900 cm^2/L. Each point represents mean and error bars show standard deviation of three biological replicates.

Table S1. Parameters in the kinetic model (30°C) with 95% confidence intervals (2)

	Value (95% Conf. Interval)				
**Parameter**	**WT**	**TS**	**TSP**	**TSPNP**	**Units**
K_a	0.0290 (0.0238, 0.0358)	0.00196 (0.0017, 0.00239)	0.00579 (0.00464, 0.00734)	0.00847 (0.00713, 0.0101)	nM^{-1}
Γ	0.0291 (0.0259, 0.0333)	0.141 (0.128, 0.153)	0.0859 (0.0679, 0.110)	0.0701 (0.0624, 0.0803)	nmol cm^{-2}
K_c	0.890 (0.861, 0.915)	20.4 (20.3, 20.4)	6.71 (5.92, 7.47)	6.89 (6.79, 6.98)	–
$k_{cat,uc}$	0.445 (0.405, 0.484)	1.11 (1.04, 1.17)	0.932 (0.738, 1.13)	1.01 (0.896, 1.13)	mA_{260} min^{-1} nM^{-1}
$k_{cat,c}$	0.0588 (0.0409, 0.0795)	0.00441 (0.001, 0.018)	0.0386 (0.0237, 0.0578)	0.0555 (0.0432, 0.0685)	mA_{260} min^{-1} nM^{-1}

Table S2. Parameters in the kinetic model (55°C) with 95% confidence intervals (2)

	Value (95% Conf. Interval)			
**Variable**	**TS**	**TSP**	**TSPNP**	**Units**
K_a	0.00793 (0.00344, 0.0139)	0.00832 (0.00284, 0.0182)	0.00869 (0.00267, 0.0187)	nM^{-1}
Γ	0.214 (0.130, 0.385)	0.228 (0.125, 0.434)	0.269 (0.192, 0.432)	nmol cm^{-2}
K_c	21.2 (20.0, 22.4)	22.8 (20.5, 25.1)	22.5 (21.2, 23.8)	–
$k_{cat,uc}$	3.77 (2.87, 4.99)	4.71 (3.43, 6.39)	4.23 (3.77, 4.71)	mA_{260} min^{-1} nM^{-1}
$k_{cat,c}$	0.489 (0.287, 0.784)	0.582 (0.328, 1.00)	0.492 (0.337, 0.646)	mA_{260} min^{-1} nM^{-1}

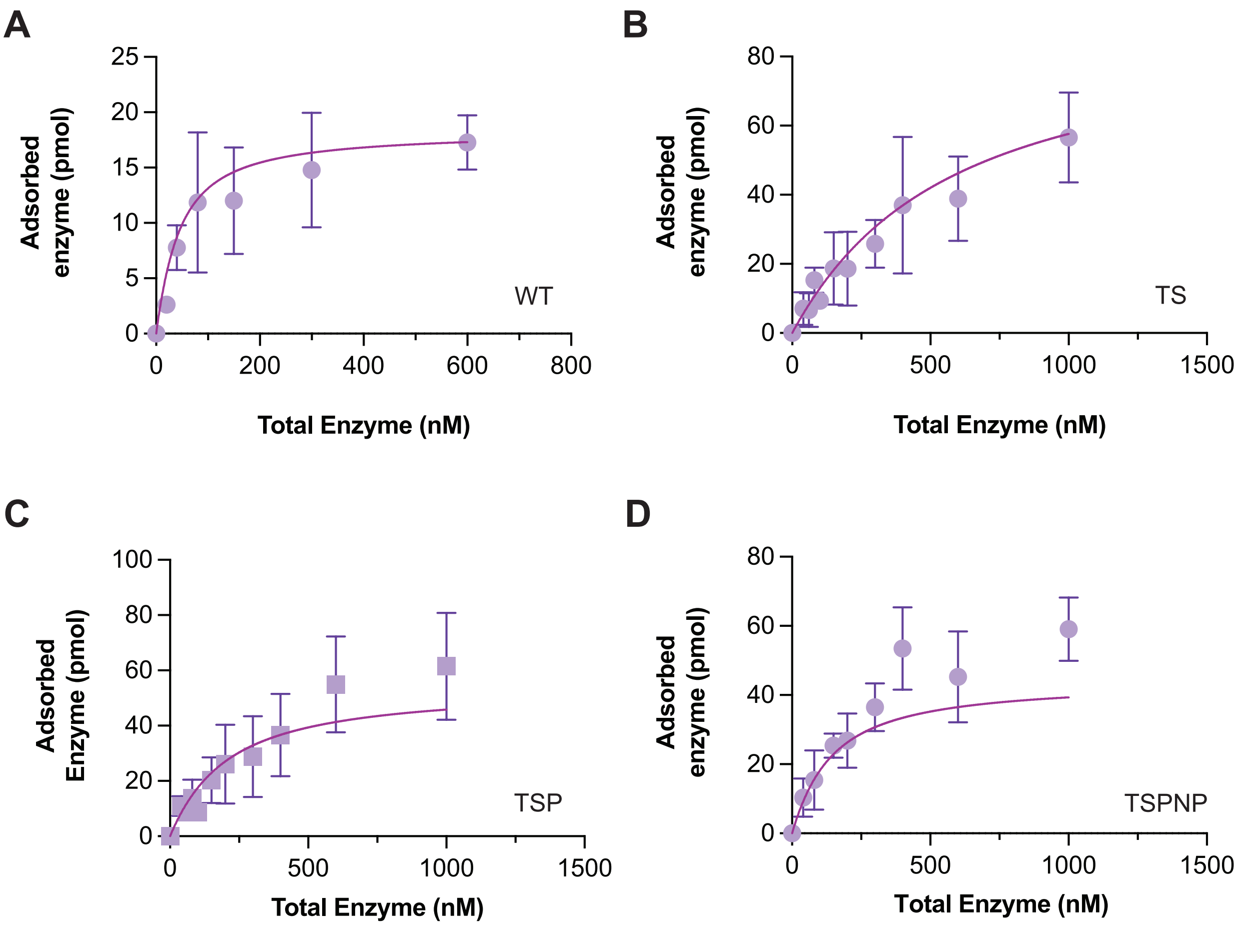

Figure S10. SC model predicted enzyme adsorption to ¼"-diameter PET films based on SC fits to kinetic data at 30°C. SC model predicted enzyme adsorption is shown in magenta curve. Mean and standard deviation are shown for experimentally measured adsorption to ¼"-diameter PET films (1 mL reaction) using solution depletion experiments ($n \geq 3$ biological replicates).

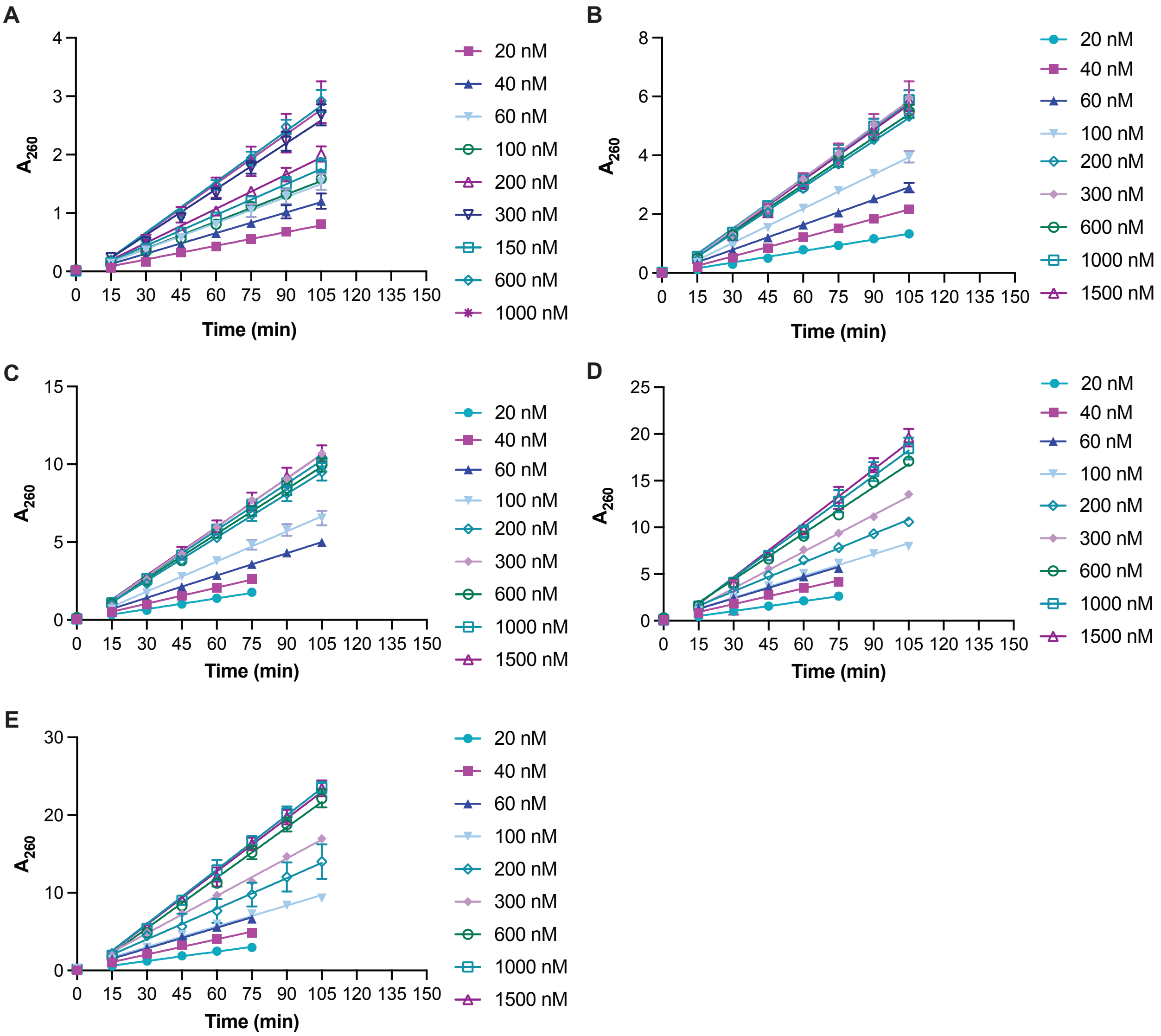

Figure S11. Example raw A_{260} values measured for TSP-PETase at 55°C within the first 105 minutes. All inital rates are calculated based on the slope between 15 and 105 minutes. Substrate concentrations shown are A) 200 cm^2/L, B) 500 cm^2/L, C) 1000 cm^2/L, D) 1500 cm^2/L, E) 1900 cm^2/L. Mean and standard deviation are shown (n = 3 biological replicates).

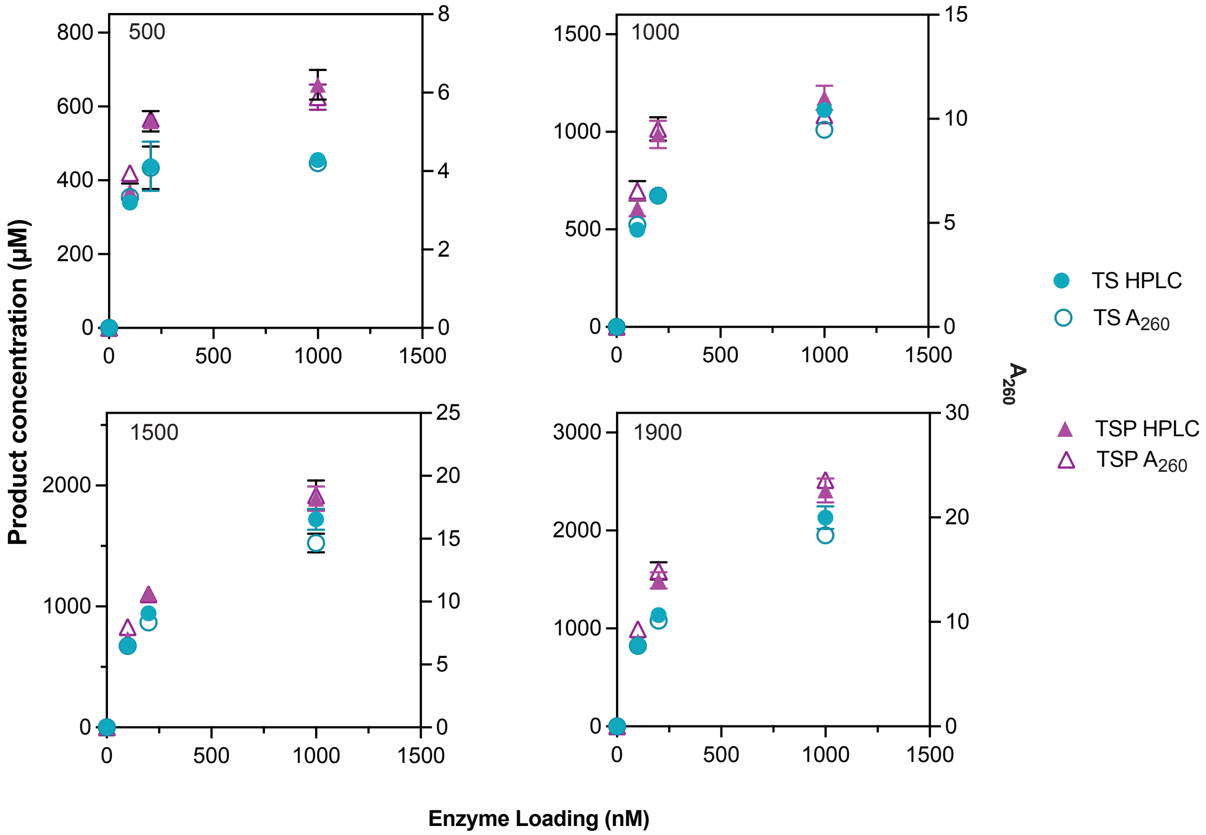

Figure S12. Comparison of HPLC product quantification with relative A_{260} measurements at 55°C. Enzyme reactions were performed over a variety of substrate concentrations, and HPLC measurements were taken at 105 minutes. Total HPLC product was reported as the sum of MHET and TPA. Four representative substrate concentrations are shown: 200, 500, 1500, and 1900 cm^2/L. Plots show mean and standard deviation of three biological replicates. A_{260} error bars are shown in black for each variant.

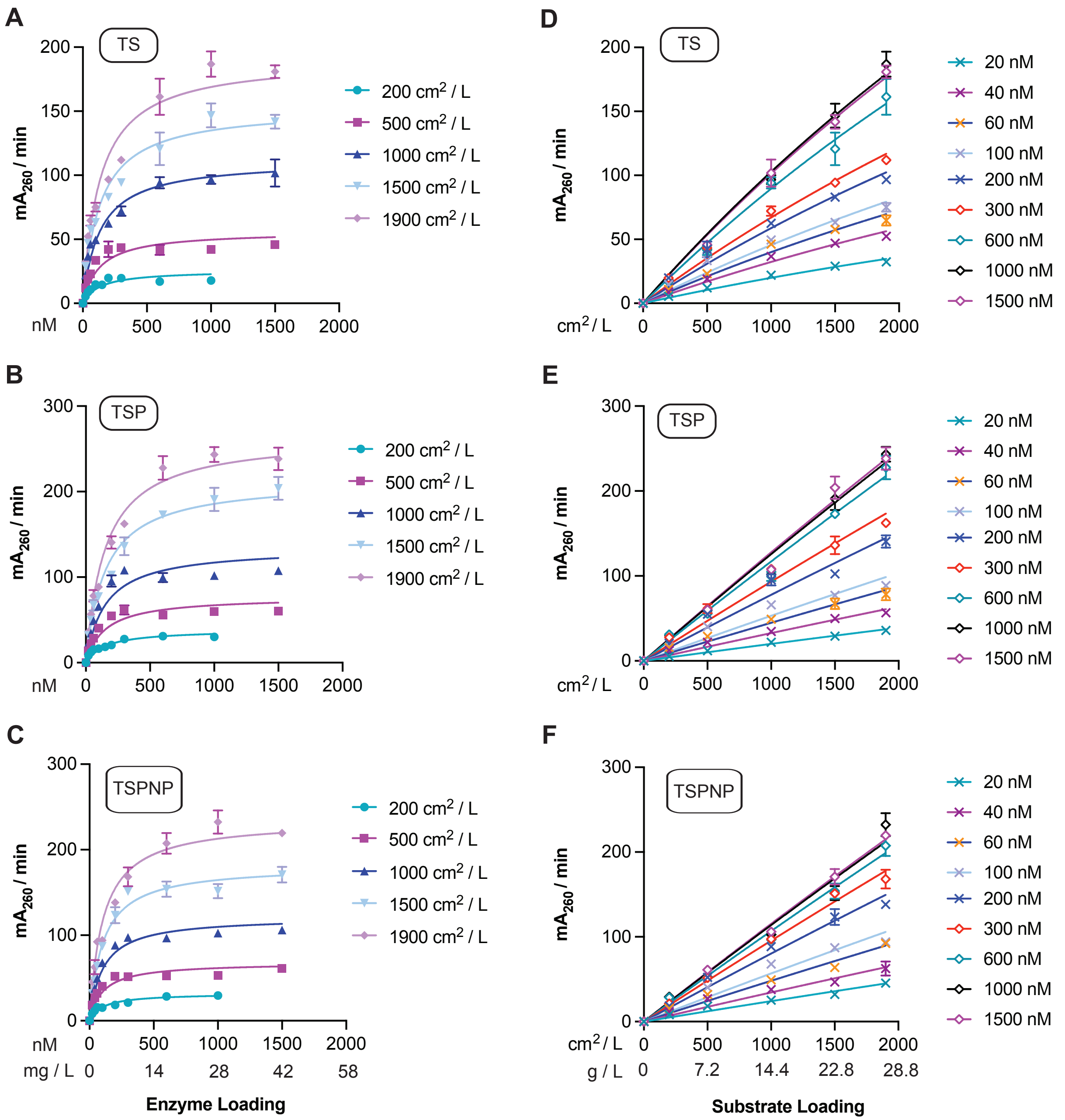

Figure S13. Fit of 55°C kinetic data with A-C) ^{inv}MM and D-F) ^{conv}MM models. Note that the fits were performed to constrain K_M to be constant across all substrate concentrations (A-C) and enzyme concentrations (D-F) as was done for fitting the same data set using the SC model. Mean and standard deviation of three biological replicates are shown.

Table S3. Rate experiments at 55°C: surface crowding (SC) model vs. inverse Michaelis-Menten (^{inv}MM) model. The table reports the maximum log-likelihood ($\hat{L}$) and the corresponding value of the Bayesian-Schwarz information criterion (BIC) for the two models. The BIC is calculated for each mutant as(3) $BIC = N_\theta \log(N_y) - 2\hat{L}$, where N_θ is the number of model parameters, N_y is the number of experimental measurements, and $\hat{L}$ is the log-likelihood calculated through the estimated parameters $\hat{\boldsymbol{\theta}}$: $\hat{L} = -\frac{1}{2}(\mathbf{y} - \tilde{\mathbf{y}}(\hat{\boldsymbol{\theta}}))^T \boldsymbol{V}_{\boldsymbol{\epsilon}}^{-1}(\mathbf{y} - \tilde{\mathbf{y}}(\hat{\boldsymbol{\theta}}))$. The SC model provides a better fit of the experimental data in terms of maximum likelihood. At the same time, the BIC computed for the SC model is lower than for the MM model despite the larger number of parameters of the SC model ($N_\theta = 5$ for the SC model, $N_\theta = 2$ for MM model). This indicates that the improved model fit from the SC model overcomes the penalty incurred from its additional parameters and is therefore a more appropriate model for the 55°C rate data.

	TS		TSP		TSPNP	
	SC	MM	SC	MM	SC	MM
$\hat{L}$	-78.02	-287.93	-214.19	-339.74	-198.96	-231.48
BIC	181.06	585.88	453.39	689.49	422.93	472.97

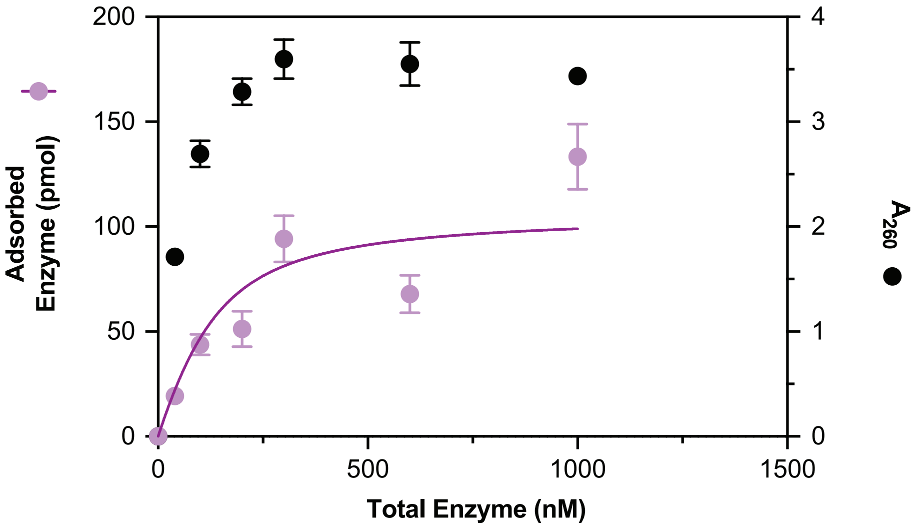

Figure S14. Adsorption of TSP-SulfoCy5 to ¼"-diameter PET film (630 cm^2/L) at 55°C based on solution depletion experiments. Difference in fluorescence measurements at 646/672 nm between PET and no PET supernatant samples after 45 minutes of incubation at 55°C and 200 rpm was used to determine enzyme adsorption. Tagged TSP-SulfoCy5 was used because it showed the most similar adsorption to TSP-PETase at 30°C; TSP-PETase could not be used due to the large amount of products formed at 55°C. Mean and standard deviation are shown for three biological replicates. Magenta curve shows the SC model predicted adsorption based on fits to 55°C kinetic data.

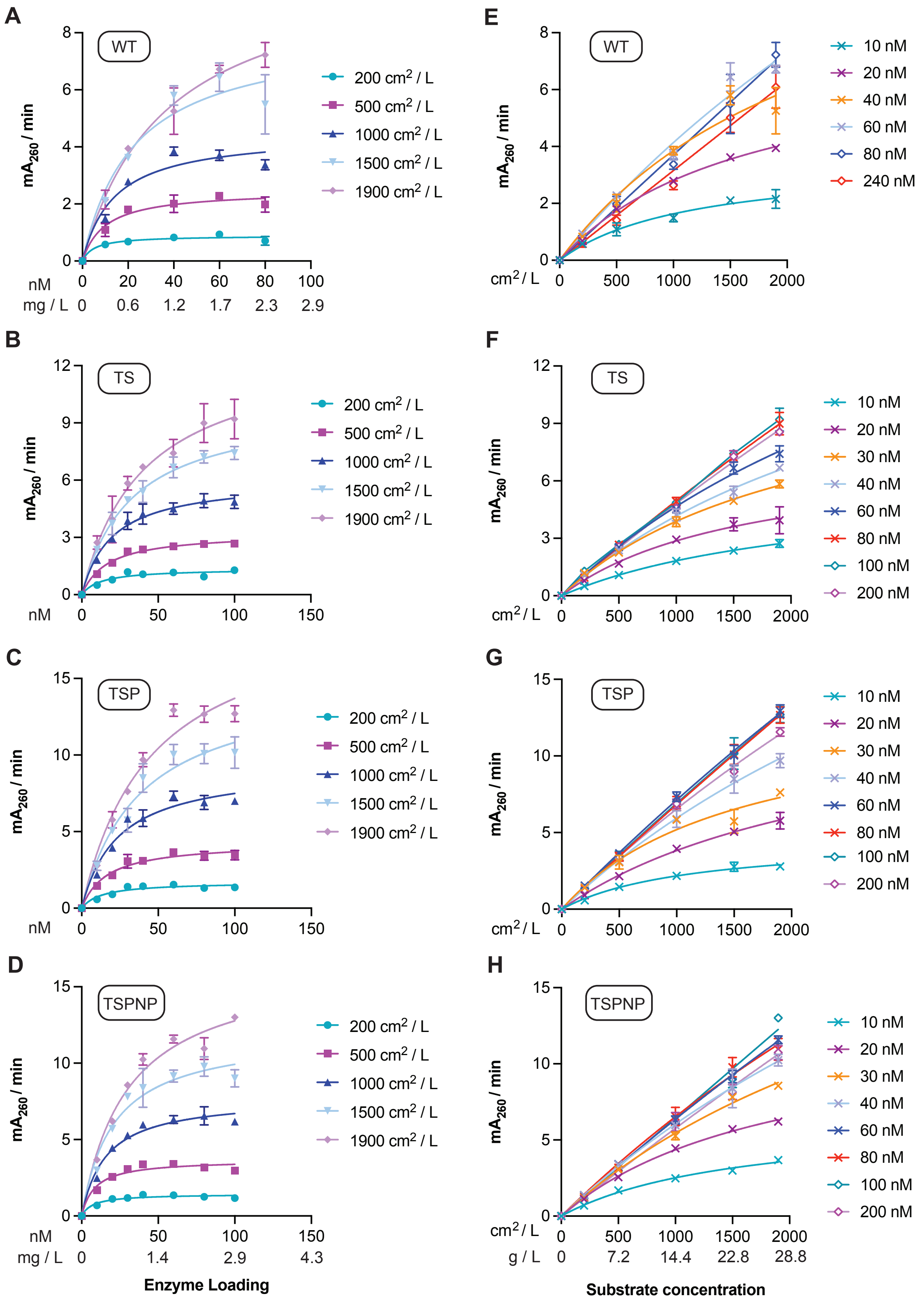
A
WT
mA260 / min
200 cm2 / L
500 cm2 / L
1000 cm2 / L
1500 cm2 / L
1900 cm2 / L
nM
mg / L
E
WT
10 nM
20 nM
40 nM
60 nM
80 nM
240 nM
cm2 / L
B
TS
F
TS
30 nM
100 nM
200 nM
C
TSP
G
TSP
D
TSPNP
H
TSPNP
g / L
Enzyme Loading
Substrate concentration

Figure S15. Fit of ^{inv}MM (A-D) and ^{conv}MM (E-H) to rate vs. enzyme concentration and rate vs. substrate concentration data for variants of *Is*PETase at 30°C. Rate vs. enzyme concentration data were truncated to remove portions of data displaying inhibition effects. Note that each substrate (A-D) or enzyme concentration (E-H) was independently fitted. Each point represents mean and error bars show standard deviation of three biological replicates.

Table S4. Fitted constants at 30°C using ^{inv}MM model for rate vs. $[E]$ and ^{conv}MM for rate vs. $[N_0]$. K is inverse of K_M for easy comparison with other models.

	**Rate vs. [*E*]** $[N_0]$ (cm^2/L)							**Rate vs. [*N*₀]** $[E]$ (nM)								
**Variant**	**Constant**	200	500	1000	1500	1900	Mean ± St. dev	**Constant**	10	20	30	40	60	80	Mean ± St. dev	$\mathbf{{}^{SA}\Gamma_{kin}}$
**WT**	${}^{SA}k_{cat}$	4.4	4.9	4.5	5.3	5.6	5.0 ± 0.60	k_{cat}	0.35	0.38		0.33	0.51	7.2e^{12}	0.35 ± 0.024	0.014
	K	0.19	0.10	0.071	0.044	0.027	0.087 ± 0.064	${}^{SA}K$ ($\times10^{-3}$)	0.87	0.60		0.42	0.15	N/A	0.63 ± 0.23	0.0072
**TS**	${}^{SA}k_{cat}$	6.9	6.1	5.8	6.4	6.6	6.3 ± 0.44	k_{cat}	0.59	0.38	0.42	0.46	0.41	1.1	0.46 ± 0.094	0.014
	K	0.076	0.072	0.057	0.034	0.027	0.053 ± 0.022	${}^{SA}K$ ($\times10^{-3}$)	0.45	0.61	0.45	0.29	0.24	0.059	0.45 ± 0.013	0.0084
**TSP**	${}^{SA}k_{cat}$	10.5	9.5	10.0	11.4	12.6	10.8 ± 1.2	k_{cat}	0.46	0.69	0.44	0.91	1.4	3.2	0.63 ± 0.23	0.017
	K	0.038	0.044	0.035	0.021	0.016	0.031 ± 0.012	${}^{SA}K$ ($\times10^{-3}$)	0.88	0.38	0.65	0.20	0.0070	N/A	0.53 ± 0.29	0.017
**TSPNP**	${}^{SA}k_{cat}$	7.2	7.3	7.8	8.0	8.7	7.8 ± 0.62	k_{cat}	0.65	0.64	0.91	1.0	1.8	0.73	0.81 ± 0.21	0.0096
	K	0.13	0.12	0.063	0.048	0.033	0.079 ± 0.044	${}^{SA}K$ ($\times10^{-3}$)	0.64	0.52	0.25	0.17	N/A	0.13	0.40 ± 0.23	0.0050

Only values fitted from 10-40 nM of enzyme were used in mean and standard deviation calculations.
${}^{SA}\Gamma_{kin}$ (nmol/L) is the kinetic surface site density factor according to Kari *et al.* (2017)(4)
${}^{SA}k_{cat} = k_{cat}({}^{SA}\Gamma_{kin})$, ${}^{SA}K = K({}^{SA}\Gamma_{kin})$, $K = 1/K_M$
Units for ${}^{SA}K$ are in L cm^{-2}, K in nM^{-1}, k_{cat} in mA$_{260}$ nM^{-1} min^{-1}, and ${}^{SA}k_{cat}$ in µA$_{260}$ L cm^{-2} min^{-1}.

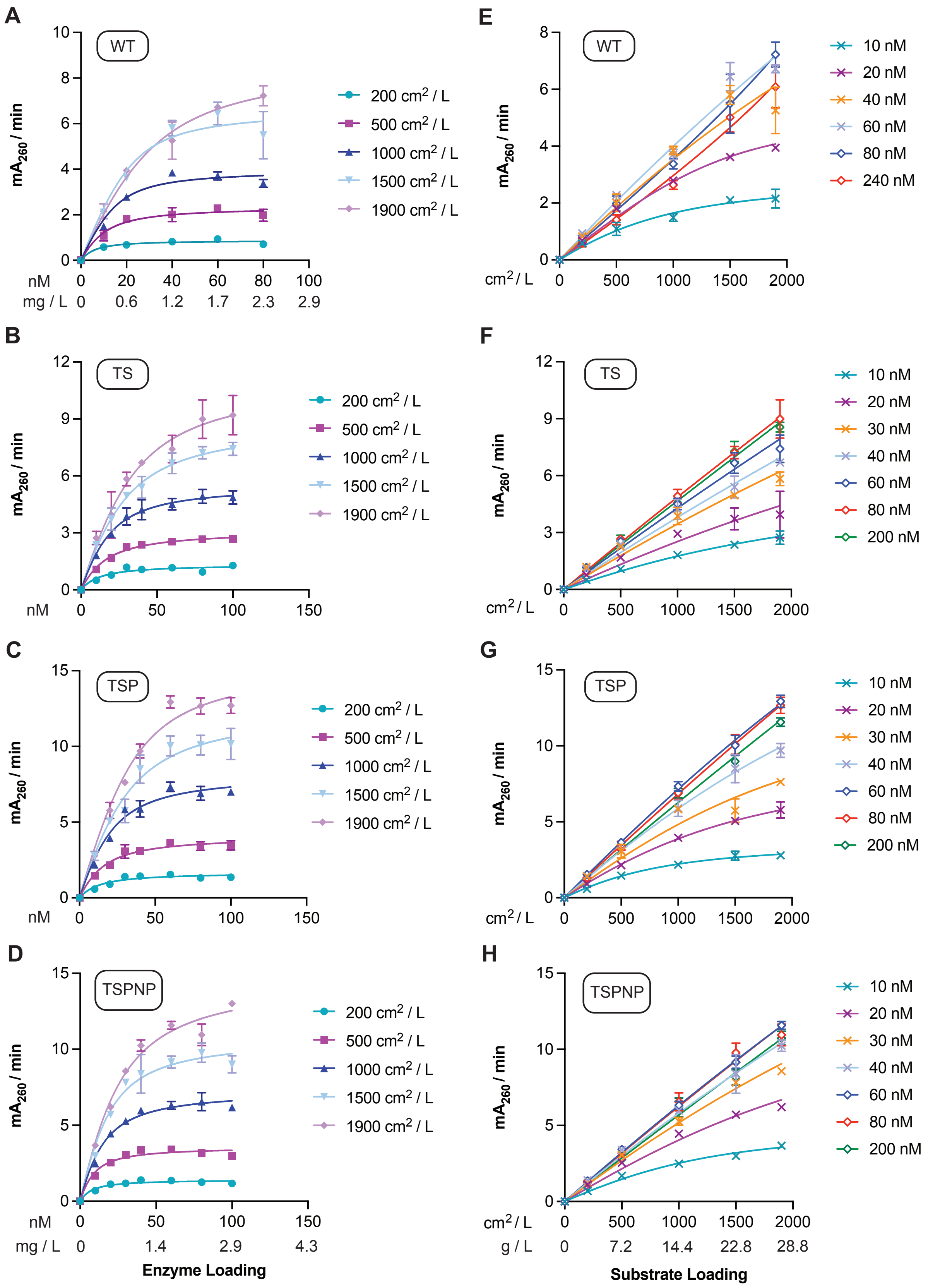
A
WT
mA260 / min
200 cm2 / L
500 cm2 / L
1000 cm2 / L
1500 cm2 / L
1900 cm2 / L
nM 0 20 40 60 80 100
mg / L 0 0.6 1.2 1.7 2.3 2.9
B
TS
nM 0 50 100 150
C
TSP
D
TSPNP
mg / L 0 1.4 2.9 4.3
Enzyme Loading
E
WT
10 nM
20 nM
40 nM
60 nM
80 nM
240 nM
cm2 / L 0 500 1000 1500 2000
F
TS
30 nM
200 nM
G
TSP
H
TSPNP
g / L 0 7.2 14.4 22.8 28.8
Substrate Loading

Figure S16. Fit of the General Michaelis Menten Equation to rate vs. enzyme concentration (A-D) and rate vs. substrate concentration data for wild-type and variants of *Is*PETase at 30°C. Rate vs. enzyme concentration data were truncated to remove portions of data displaying inhibition effects. Note that each substrate (A-D) or enzyme concentration (E-H) was independently fitted. Each point represents mean and error bars show standard deviation of three biological replicates.

Table S5. Fitted k_{cat} and K constants at 30°C using the General Michaelis-Menten model. K is inverse of K_M for easy comparison with other models.

		**Rate vs. [E]** [N_0] (cm^2/L)					**Rate vs. [N_0]** [E] (nM)							
**Variant**	**Constant**	200	500	1000	1500	1900	10	20	30	40	60	80	200	Mean ± St. dev
**WT**	k_{cat}	0.33	0.34	0.28	0.32	0.33	0.29	0.26		0.40	0.49	0.26*		0.34 ± 0.071
	K	0.13	0.15	0.22	0.20	0.082	0.16	0.32		0.054	0.026	$1.8e^{12*}$		0.15 ± 0.091
**TS**	k_{cat}	0.48	0.44	0.39	0.42	0.42	0.50	0.24	0.42	0.49	0.56	0.67	0.64	0.48 ± 0.085
	K	0.094	0.085	0.10	0.068	0.062	0.056	0.57	0.076	0.043	0.025	0.015	0.006	0.062 ± 0.028
**TSP**	k_{cat}	0.54	0.49	0.48	0.49	0.48	0.38	0.46	0.43	0.52	0.54	0.45	0.71	0.48 ± 0.047
	K	0.054	0.078	0.10	0.071	0.087	0.13	0.085	0.10	0.069	0.074	0.14	0.006	0.089 ± 0.026
**TSPNP**	k_{cat}	0.71	0.71	0.73	0.72	0.77	0.50	0.61	0.86	1.1	1.2	1.2	1.1	0.82 ± 0.23
	K	0.14	0.13	0.081	0.067	0.046	0.21	0.15	0.063	0.036	0.019	0.016	0.005	0.088 ± 0.063

* Only values fitted from 10-80 nM of enzyme were used in mean and standard deviation calculations from rate vs. [S] data. Additional excluded values are marked by [*]. Mean and standard deviation are calculated across each row.

Units for K are in nM^{-1}, k_{cat} in mA_{260} nM^{-1} min^{-1}.

TSPNP = TSP-S238N-S290P

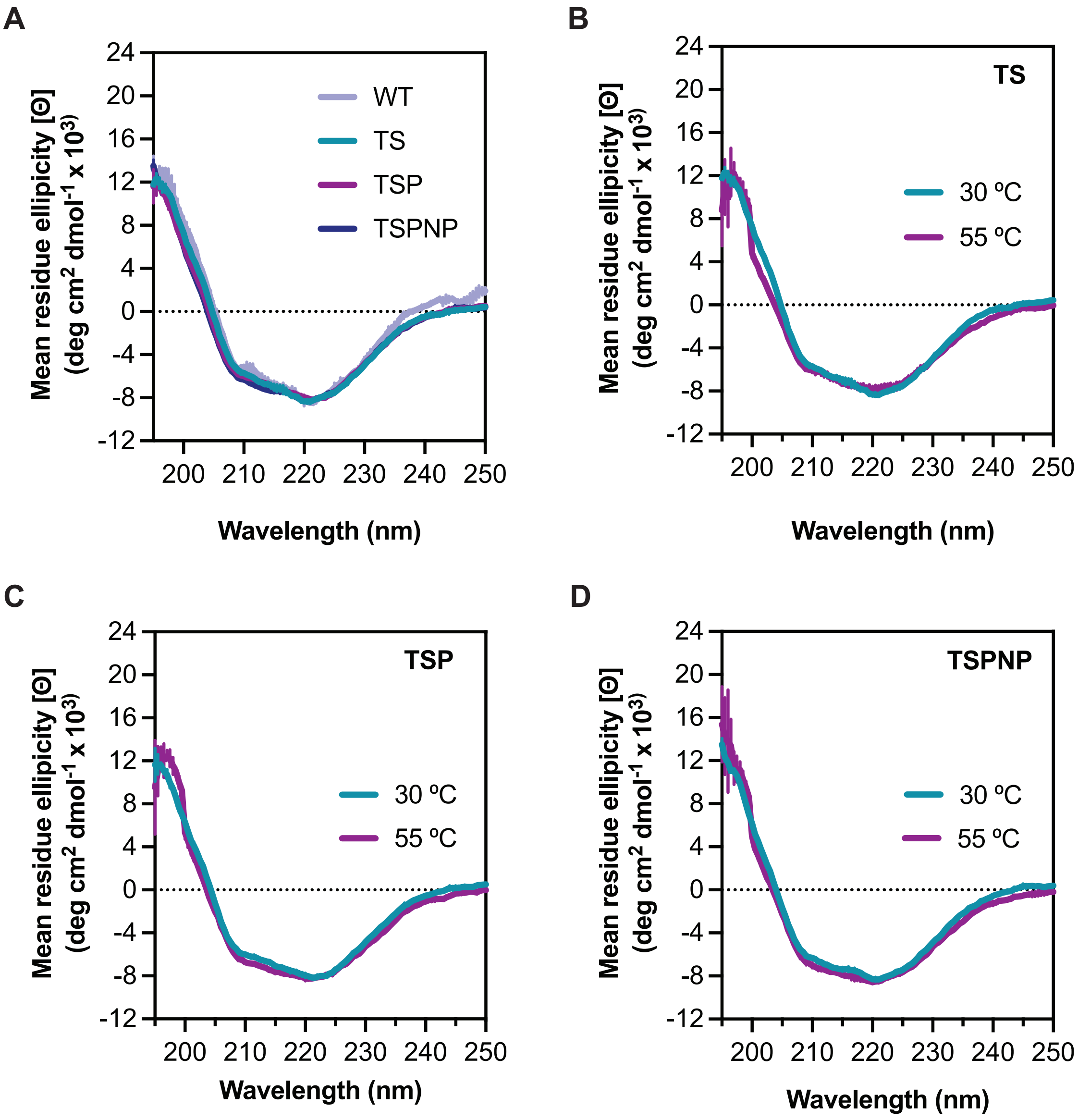

Figure S17. Circular dichroism (CD) spectrometry of *Is*PETase variants. A) CD measurements of WT *Is*PETase (WT), TS-PETase (TS), TSP-PETase (TSP), and TSPNP-PETase (TSPNP) at 30°C. CD measurements of B) TS-PETase, C) TSP-PETase, and D) TSPNP-PETase at 55°C compared to 30°C. All curves and error bars are mean and standard deviation of three technical replicates, respectively. Samples were diluted to 0.075 mg/mL and dialyzed into 20 mM phosphate pH 7.2, 50 mM NaCl and data collected on a JASCO J-1500-150 CD Spectrometer.

Primers	Sequence
pET21a_F	CTCGAGCACCACCAC
pET21a_R	ATGTATATCTCCTTCTTAAAGTTAAACA
IsPETase_F	TTTAAGAAGGAGATATACATATGCAGACCAATCCGTATGCA
IsPETase_R	TGGTGGTGGTGGTGCTCGAGGCTACAATTTGCGGTACGAA AA
pET21a_His$_8$_F	CACTGAGATCCGGCTGCTAACAAAG
pET21a_His$_8$_R	CTTTGTTAGCAGCCGGATCTCAGTGGTGGTGGTGGTGGTG GTGGTGCTCGAGGCTACAATT
IsPETase_mut_F	GTTTAACTTTAAGAAGGAGATATACATATG
IsPETase_mut_R	GTGGTGGTGGTGCTCGAG
T116P_F	ACCAACAGCCCGCTGGATCAGCCGGAAAG
T116P_R	CAGGCCGCTGTTGGTATCAATGGTAATAAC
S238N_F	GCAGCCATAATTGTGCAAATAGCGGTAATAGC
S238N_R	TTTGCACAATTATGGCTGCCACCACAAATTTC
T88L_F	CGGGTTATCTGGCACGTCAGAGCAGCATTAA
T88L_R	TGACGTGCCAGATAACCCGGAACAATGGCAATT
S290P_F	CAAATTGTCCGCTCGAGCACCACCAC
S290P(S282C)_R	TGCTCGAGCGGACAATTTGCGGTACGAAAATCACAAAC
pET21a_Srt_F	CTGCCGGAAACCGGCGGCCTCGAGCACCACCACCACCAC CACTGAG
IsPETase_Srt_R	GCCGCCGGTTTCCGGCAGGCTACAATTTGCGGTACGAA AATC

Table S6. Primers used to clone all plasmids used in this study.

Supplementary Methods

*Is*PETase DNA sequences

WT *Is*PETase-His$_6$:

ATGCAGACCAATCCGTATGCACGTGGTCCGAATCCGACCGCAGCAAGCCT
GGAAGCAAGCGCAGGTCCGTTTACCGTTCGTAGCTTTACCGTTAGCCGTC
CGAGCGGTTATGGTGCAGGCACCGTTTATTATCCGACCAATGCCGGTGGC
ACCGTTGGTGCAATTGCCATTGTTCCGGGTTATACCGCACGTCAGAGCAG
CATTAAATGGTGGGGTCCGCGTCTGGCAAGCCATGGTTTTGTTGTTATTA
CCATTGATACCAACAGCACCCTGGATCAGCCGAGCAGCCGTAGCAGTCAG
CAGATGGCAGCACTGCGTCAGGTTGCCAGCCTGAATGGCACCAGCAGCAG
CCCGATTTATGGTAAAGTTGATACAGCACGTATGGGTGTTATGGGTTGGA
GCATGGGTGGTGGTGGTAGCCTGATTAGTGCAGCAAATAATCCGAGCCTG
AAAGCAGCCGCACCGCAGGCTCCGTGGGATAGCAGCACCAATTTTAGCAG
CGTTACCGTTCCGACACTGATTTTTGCATGTGAAAATGATAGCATTGCAC
CGGTTAATAGCAGCGCACTGCCGATCTATGATAGTATGAGCCGTAATGCA
AAACAGTTTCTGGAAATTAATGGTGGCAGCCATAGCTGTGCAAATAGCGG
TAATAGCAATCAGGCACTGATCGGTAAAAAAGGTGTTGCATGGATGAAAC
GCTTCATGGATAATGATACCCGTTATAGCACCTTTGCCTGCGAAAATCCG
AATAGCACCCGTGTTAGCGATTTTCGTACCGCAAATTGTAGCCTCGAGCA
CCACCACCACCACCACTGA

TS-PETase-His$_6$:

ATGCAGACCAATCCGTATGCACGTGGTCCGAATCCGACCGCAGCAAGCCT
GGAAGCAAGCGCAGGTCCGTTTACCGTTCGTAGCTTTACCGTTAGCCGTC
CGAGCGGTTATGGTGCAGGCACCGTTTATTATCCGACCAATGCCGGTGGC
ACCGTTGGTGCAATTGCCATTGTTCCGGGTTATACCGCACGTCAGAGCAG
CATTAAATGGTGGGGTCCGCGTCTGGCAAGCCATGGTTTTGTTGTTATTA
CCATTGATACCAACAGCACCCTGGATCAGCCGGAAAGCCGTAGCAGTCAG
CAGATGGCAGCACTGCGTCAGGTTGCCAGCCTGAATGGCACCAGCAGCAG
CCCGATTTATGGTAAAGTTGATACAGCACGTATGGGTGTTATGGGTTGGA

GCATGGGTGGTGGTGGTAGCCTGATTAGTGCAGCAAATAATCCGAGCCTG
AAAGCAGCCGCACCGCAGGCTCCGTGGCATAGCAGCACCAATTTTAGCAG
CGTTACCGTTCCGACACTGATTTTTGCATGTGAAAATGATAGCATTGCAC
CGGTTAATAGCAGCGCACTGCCGATCTATGATAGTATGAGCCGTAATGCA
AAACAGTTTCTGGAAATTTGTGGTGGCAGCCATAGCTGTGCAAATAGCGG
TAATAGCAATCAGGCACTGATCGGTAAAAAAGGTGTTGCATGGATGAAAC
GCTTCATGGATAATGATACCCGTTATAGCACCTTTGCCTGCGAAAATCCG
AATAGCACCGCTGTTTGTGATTTTCGTACCGCAAATTGTAGCCTCGAGCA
CCACCACCACCACCACTGA

TSP-PETase-His$_6$:

ATGCAGACCAATCCGTATGCACGTGGTCCGAATCCGACCGCAGCAAGCCT
GGAAGCAAGCGCAGGTCCGTTTACCGTTCGTAGCTTTACCGTTAGCCGTC
CGAGCGGTTATGGTGCAGGCACCGTTTATTATCCGACCAATGCCGGTGGC
ACCGTTGGTGCAATTGCCATTGTTCCGGGTTATACCGCACGTCAGAGCAG
CATTAAATGGTGGGGTCCGCGTCTGGCAAGCCATGGTTTTGTTGTTATTA
CCATTGATACCAACAGCCCGCTGGATCAGCCGGAAAGCCGTAGCAGTCAG
CAGATGGCAGCACTGCGTCAGGTTGCCAGCCTGAATGGCACCAGCAGCAG
CCCGATTTATGGTAAAGTTGATACAGCACGTATGGGTGTTATGGGTTGGA
GCATGGGTGGTGGTGGTAGCCTGATTAGTGCAGCAAATAATCCGAGCCTG
AAAGCAGCCGCACCGCAGGCTCCGTGGCATAGCAGCACCAATTTTAGCAG
CGTTACCGTTCCGACACTGATTTTTGCATGTGAAAATGATAGCATTGCAC
CGGTTAATAGCAGCGCACTGCCGATCTATGATAGTATGAGCCGTAATGCA
AAACAGTTTCTGGAAATTTGTGGTGGCAGCCATAGCTGTGCAAATAGCGG
TAATAGCAATCAGGCACTGATCGGTAAAAAAGGTGTTGCATGGATGAAAC
GCTTCATGGATAATGATACCCGTTATAGCACCTTTGCCTGCGAAAATCCG
AATAGCACCGCTGTTTGTGATTTTCGTACCGCAAATTGTAGCCTCGAGCA
CCACCACCACCACCACTGA

TSPNP-PETase-His$_6$:

ATGCAGACCAATCCGTATGCACGTGGTCCGAATCCGACCGCAGCAAGCCT
GGAAGCAAGCGCAGGTCCGTTTACCGTTCGTAGCTTTACCGTTAGCCGTC
CGAGCGGTTATGGTGCAGGCACCGTTTATTATCCGACCAATGCCGGTGGC
ACCGTTGGTGCAATTGCCATTGTTCCGGGTTATACCGCACGTCAGAGCAG
CATTAAATGGTGGGGTCCGCGTCTGGCAAGCCATGGTTTTGTTGTTATTA
CCATTGATACCAACAGCCCGCTGGATCAGCCGGAAAGCCGTAGCAGTCAG
CAGATGGCAGCACTGCGTCAGGTTGCCAGCCTGAATGGCACCAGCAGCAG
CCCGATTTATGGTAAAGTTGATACAGCACGTATGGGTGTTATGGGTTGGA
GCATGGGTGGTGGTGGTAGCCTGATTAGTGCAGCAAATAATCCGAGCCTG
AAAGCAGCCGCACCGCAGGCTCCGTGGCATAGCAGCACCAATTTTAGCAG
CGTTACCGTTCCGACACTGATTTTTGCATGTGAAAATGATAGCATTGCAC
CGGTTAATAGCAGCGCACTGCCGATCTATGATAGTATGAGCCGTAATGCA
AAACAGTTTCTGGAAATTTGTGGTGGCAGCCATAATTGTGCAAATAGCGG
TAATAGCAATCAGGCACTGATCGGTAAAAAAGGTGTTGCATGGATGAAAC
GCTTCATGGATAATGATACCCGTTATAGCACCTTTGCCTGCGAAAATCCG
AATAGCACCGCTGTTTGTGATTTTCGTACCGCAAATTGTCCGCTCGAGCA
CCACCACCACCACCACTGA

In silico directed evolution

Computational prediction of long-range mutations. A thorough computational screening was performed to find distal mutations able to improve TSPETase properties. Our approach utilized Zymvol's in-house algorithm, Zymevolver, which leverages bioinformatics and structure-based tools to pinpoint distal enzyme hotspots with the potential to enhance enzyme catalytic properties. This approach effectively identifies distal enzyme hotspots that may impact the prevalence of catalytically relevant productive enzyme conformations. The method has been successfully applied in over 20 enzyme engineering campaigns and has consistently demonstrated its remarkable efficacy in boosting enzyme activity, delivering single mutants with activity improvements up to 46-fold. In this particular study, Zymevolver identified 46 hotspots, covering 13% of the protein, within the previously prepared TS-PETase structure. We assessed the amino acid variability at each hotspot, guided by phylogenetic and structural information, which conducted the selection of 42 distal single-point variants. Furthermore, we employed two publicly available web servers, FireProt and PROSS, for the automatic generation of thermostable variants by using sequence and structural information(5, 6). Generated variants were subjected to a comprehensive evaluation and ranking process, considering various

computational determinants such as free energy and hotspot mutability. Ultimately, 13 additional multiple-point variants were selected for experimental validation.

Active site engineering. A PET dimer model was bound into the active site of the TS-PETase previously prepared structure using Autodock VINA (7). The simulation box was centered on the TS-PETase catalytic triad residues, namely S160, H237, and D206, and extended 8 Å from these catalytic positions. After docking, the top 10 docking poses that showed a pre-catalytic configuration were selected to be used as starting point for the subsequent Rosetta Coupled Moves mutagenesis experiments (8). In these mutagenesis experiments, strategically chosen non-catalytic active site residues in proximity to the bound PET dimer were selected for mutagenesis, including residues 85, 88, 89, 112, 118, 123, 183, 186, 207, 208, 209, 236, 238, and 241 (Figure S2D). The Rosetta Coupled Moves Monte Carlo algorithm facilitated simultaneous exploration of changes in one or two amino acid positions, generating a library of single and double mutants. Residue repacking was restricted to only those residues in direct contact with the mutated positions. A ligand weight of 1.0 and a Boltzmann constant of 0.6 were applied, and each experiment consisted of 100 Coupled Moves runs of 1000 trials. The resulting mutant scaffolds from Rosetta were then filtered using protein-ligand docking and 0.5 ns Molecular Dynamic (MD) simulations to evaluate whether the virtual mutants contributed to the stabilization of the PET dimer ligand. Multiple criteria, including enzyme-substrate interactions, the prevalence of pre-catalytic configurations during MD simulations, Rosetta TotalScore, LigandScore, and docking binding energies, were employed to curate a final selection of 40 variants.

High-throughput screening of *Is*PETase variants

Competent *E. coli* T7 Express were transformed with directed evolution libraries using heat shock: 30 minutes on ice and 25 seconds at 42°C. The cells were recovered in LB for 30 minutes at 37°C, and plated onto large LB+100 µg/mL Carbenicillen (Carb) agar plates. Transformants were cultured in 50 µL of LB+Carb in 384-well plates overnight at 37°C and 250 rpm, and glycerol stocks were prepared by adding 50 µL of 40% glycerol to all wells and then stored at -80°C. Library starter cultures were grown overnight at 37°C in 200 µL of LB+Carb in 2-mL 96-well plates shaken at 600 rpm in a ThermoMixer (Eppendorf). Starter cultures were inoculated 1:100 into either 1.8 mL (wild-type library) or 0.5 mL (TSP-PETase library) of Terrific Broth+Carb (TB+Carb) and grown for 4 hours at 37°C (750 rpm). PETase expression was induced with 1 mM IPTG and incubated at 16°C for 24 hours shaking at 750 rpm. Plates were spun down at 4000 rpm for 20 minutes and medium was removed. Lysis buffer (0.043 g/mL CelLytic Express powder (Millipore Sigma) in wash buffer (50 mM glycine pH 9, 50 mM NaCl, 40 mM imidazole buffer)) was added to the cell pellets (300µL/well for WT library, 150 µL/well for TSP-PETase library) and incubated at 25°C for

10 minutes at 900 rpm followed by 10 minutes at 400 rpm. The lysate was centrifuged at 4000 rpm for 20 minutes, and supernatant was transferred to a new 2-mL 96-well plate. 3 µL of lysate was spotted onto a rectangular BHET-agar plate (6 mg/mL BHET suspended in 2% agar buffered with 100 mM glycine-NaOH pH 9) and incubated at 30°C for 2-6 hours. Wells displaying zones of clearing were incubated with magnetic Ni-NTA resin (GenScript) overnight at 4°C, 900 rpm (25 µL/well of magnetic slurry for WT library, 15 µL/well of magnetic slurry for TSP-PETase library). Supernatant with resin was transferred to a PCR plate fitted on top of a 96-well ring magnet (96S Super, Alpaqua), and washed eight times with 100 µL wash buffer. After the final wash, 120 µL of elution buffer (50 mM glycine pH 9, 50 mM NaCl, 150 mM imidazole) was added to each well and incubated at room temperature for 15 minutes with intermittent mixing. MicroBCA (ThermoFisher) reagent was prepared according to directions. 40 µL of MicroBCA mixture was incubated with 8 µL of elution in duplicate for 2 hours at 37°C and protein quantified (standard curve 12.5-300 µg/mL). All samples were normalized with elution buffer to 400 nM if possible. Circular PET films (¼-inch diameter, Goodfellow USA ES30-FM-000145) were placed vertically into each well of a round-bottom 96-well plate (Greiner Bio-One). 50 µL of the normalized elution was incubated with 150 µL of reaction buffer (50 mM glycine pH 9, 50 mM NaCl, 10% v/v DMSO) for 2 hours at 30°C in the round-bottom plates at 900 rpm. 100 µL of the reaction was transferred to a UV-transparent 384-well plate (Greiner Bio-one) and absorbance at 260 nm was measured with a plate reader (BioTek). Variants showing higher protein yields/and or A_{260} readings above control were validated using the high-throughput screening protocol in triplicate or quadruplets.

Reverse-phase HPLC

High pressure liquid chromatography (HPLC) measurements were performed as previously described (mobile phase A: 20 mM phosphate pH 2.5; mobile phase B: methanol) (9). 700 µL of the reaction supernatant was taken and heated at 85°C for 15 minutes; 10 µL of sample was injected onto an Eclipse XDB-C18 column (Agilent) and quantified based on MHET and TPA standards (0-500 µM in 1:1 reaction buffer and 40 mM phosphate pH 2.5). For HPLC quantification of BHET hydrolysis in the presence of Ficoll 70, 300 µL of reaction and 300 µL of 200 mM phosphate pH 2.5 were mixed to terminate the reaction and subsequently analyzed.

TIRF microscopy

In brief, 638 nm continuous wave laser excitation is generated via a 638 nm CW laser diode (Modulated Laser Diode 638 nm, 180 mW, Cobolt). The power of the laser is set to approximately 0.9-1.0 mW at the objective. Excitation light is reflected off a dichroic (Di03-R405/488/561/635-t1-25x36, Semrock) and transmitted through an objective (APON60XOTIRF, Olympus) suitable for TIRF microscopy. Incoming excitation light is aligned and focused to totally internally reflect at the glass/sample interface to create an evanescent field which will penetrate the sample. The penetration depth was calibrated on a DNA control sample as described previously(10). Fluorescence is then collected through the objective and transmitted through the dichroic and imaged on a sCMOS camera (Orca Flash 4.0 V3, Hamamatsu). Image splitting optics (Gemini Optical Splitter, Hamamatsu) are used to create two images of the sample at two different wavelengths corresponding to the emission profiles of the dye pair used in FRET experiments via a dichroic (T647cxr-UF2, Chroma), referred to as the donor and acceptor channels henceforth. Band pass filters are used for additional spectral filtering prior to detection on the camera (FF01-679/41-25 for the acceptor channel, Semrock). The labeled enzyme was exclusively imaged in the acceptor channel. HCImage (Hamamtsu) is used for camera control and image acquisition. The camera is set to acquire 2,400 frames with a 100-millisecond integration time per frame, giving a 4-minute image acquisition.

Monte Carlo Simulations of Surface Density

To calculate the expected minimum distance between molecules on the substrate, a simple Monte-Carlo method was developed. Molecules were placed at random locations according to a uniform probability distribution with the same number of molecules as in the experimental data or predictions. The distribution of minimum distances was extracted the same way as the TIRF data. The reported values are the average minimum distances. Uncertainties are not reported due to the large number of points used in the Monte-Carlo model.

Additional information on the derivation of the biochemical model

The enzymes adsorbed on the substrate sites present a distribution of catalytic rate constants, based on the local crowding conditions:

$$\frac{dP}{dt} = \sum_{i=1}^{N} k_{cat,c,i}[EN_{c,i}], \tag{S1}$$

$$[EN] = \sum_{i=1}^{N}[EN_{c,i}], \tag{S2}$$

where $[EN_{c,i}]$ is the concentration of enzymes in crowding conditions compatible with a catalytic rate constant $k_{cat,c,i}$, N is the length of the distribution, and $[EN]$ is the total amount of adsorbed enzymes. As discussed in the main text, $[EN]$ is not affected by crowding, and is obtained through Eq. 6. Constants $k_{cat,c,i}$ are sorted in decreasing sense, with $k_{cat,c,1}$ corresponding to the maximum catalytic rate constant, associated to no crowding limitation. It immediately follows that $k_{cat,c,1}$ is the catalytic rate constant per enzyme in an ideal completely uncrowded scenario ($\theta = 0$). Since the minimum distance between two adsorbed enzymes is fixed and is related to Γ, every enzyme will present, at full site coverage ($\theta = 1$), the same catalytic rate constant $k_{cat,c,N}$, corresponding to the lower bound of the $k_{cat,c,i}$ distribution. The biochemical model of Eqs 6, 4, 17, and 18 approximates the distribution of enzyme activity by imposing $N = 2$, namely by considering the presence in the system of only (i) enzymes EN_{uc} not affected by crowding at all (catalytic rate constant $k_{cat,uc} = k_{cat,1}$) and (ii) enzymes presenting the maximum limitation that can be induced by crowding (catalytic rate constant $k_{cat,c} = k_{cat,N} = k_{cat,2}$):

$$\frac{dP}{dt} = \sum_{i=1}^{N} k_{cat,c,i}[EN_{c,i}] \approx k_{cat,uc}[EN_{uc}] + k_{cat,c}[EN_c], \tag{S3}$$

$$[EN] = \sum_{i=1}^{N} [EN_{c,i}] = [EN_{uc}] + [EN_c]. \tag{S4}$$

The catalytic rate of Eq. S3 and the final form of the equation derived in the main text (Eq. 18) effectively approximate the distribution of the catalytic rates of the enzyme population through a weighted average of the upper and lower bounds of the catalytic rate constant distribution. Following the approximation of the crowded enzyme configurations into a single species EN_c, the transition between EN_{uc} and EN_c is represented by the following reactions:

$$EN_{uc} + EN_{uc} \underset{k_{uc}}{\overset{k_c}{\rightleftharpoons}} EN_c + EN_c, \tag{S5}$$

$$EN_{uc} + EN_c \underset{k_{uc}}{\overset{k_c}{\rightleftharpoons}} EN_c + EN_c, \tag{S6}$$

which are stoichiometrically equivalent to Eq. 11. According to Eqs S5 and S6, an enzyme in uncrowded configuration EN_{uc} can transition to a crowded configuration EN_c due to a crowding interaction with another enzyme, which can be either in uncrowded or in crowded configuration. In the former case, both enzymes will assume a crowded configuration; in the latter case, the enzyme that was already in crowded configuration will not undergo an additional decrease of catalytic rate constant, since the model assumes that there is only one catalytic rate constant for all enzymes in crowded configurations. From Eqs S5–S6,

the rate of transition from uncrowded to crowded enzyme configuration (r_c) is:

$$r_c = k_c[EN_{uc}]^2 + k_c[EN_{uc}][EN_c], \tag{S7}$$

which is equivalent to Eq. 13. The rate of transition from a crowded to an uncrowded configuration depends on many factors, such as the enzyme flexibility and the distance between sites in a given substrate. Most of all, availability of free space on the substrate surface is necessary for an enzyme to rearrange into an uncrowded configuration. Hence, we represent r_{uc} as:

$$r_{uc} = k_{uc}[EN_c]([N_T] - [EN]), \tag{S8}$$

which effectively accounts for the decrease of r_{uc} as the concentration of free substrate surface decreases.

References

1. Badino, S. F., Bth, J. A., Borch, K., Jensen, K., and Westh, P. (2022) Adsorption of enzymes with hydrolytic activity on polyethylene terephthalate. *Enzyme Microb. Technol.* **152**, 109937

2. Efron, B. and Tibshirani, R. J. (1994) *An Introduction to the Bootstrap.* Boca Raton, FL: CRC Press

3. Llorente, F., Martino, L., Delgado, D., and López-Santiago, J. (2023) Marginal likelihood computation for model selection and hypothesis testing: An Extensive Review. *SIAM Rev.* **65**, 3–58

4. Kari, J., Andersen, M., Borch, K., and Westh, P. (2017) An inverse Michaelis-Menten approach for interfacial enzyme kinetics. *ACS Catal.* **7**, 4904–4914

5. Musil, M., Stourac, J., Bendl, J., Brezovsky, J., Prokop, Z., Zendulka, J., *et al.* (2017) FireProt: web server for automated design of thermostable proteins. *Nucleic Acids Res.* **45**, W393–W399

6. Goldenzweig, A., Goldsmith, M., Hill, S. E., Gertman, O., Laurino, P., Ashani, Y., *et al.* (2016) Automated structure- and sequence-based design of proteins for high bacterial expression and stability. *Mol. Cell* **63**, 337 – 346

7. Trott, O. and Olson, A. J. (2010) AutoDock Vina: Improving the speed and accuracy of docking with a new scoring function, efficient optimization, and multithreading. *J. Comput. Chem.* **31**, 455–461

8. Ollikainen, N., Jong, R. M. d., and Kortemme, T. (2015) Coupling protein side-chain and backbone flexibility improves the re-design of protein-ligand specificity. *PLoS Comput. Biol.* **11**, e1004335

9. Zhong-Johnson, E. Z. L., Voigt, C. A., and Sinskey, A. J. (2021) An absorbance method for analysis of enzymatic degradation kinetics of poly(ethylene t erephthalate) films. *Sci. Rep.* **11**, 928

10. Dresser, L., Hunter, P., Yendybayeva, F., Hargreaves, A. L., Howard, J. A., Evans, G. J., *et al.* (2021) Amyloid- oligomerization monitored by single-molecule stepwise photobleaching. *Methods* **193**, 80–95
